# Harnessing Bifunctional Nitrogen‐Dislocation Interactions for a Record Ultra‐Strong‐and‐Ductile Duplex Titanium Alloy

**DOI:** 10.1002/advs.202502349

**Published:** 2025-06-19

**Authors:** Chongle Zhang, Xuanzhe Li, Suzhi Li, Jinyu Zhang, Gang Liu, Jun Sun

**Affiliations:** ^1^ State Key Laboratory for Mechanical Behavior of Materials Xi'an Jiaotong University Xi'an 710049 P.R. China

**Keywords:** < ***c*** + ***a*** > dislocations, interstitial atoms‐dislocations interactions, low‐angle grain boundaries, mechanical properties, titanium alloys

## Abstract

Duplex (α+β) Ti alloys often manifest limited uniform elongation (ε_u_) mainly originating from the lack of < ***c*** + ***a*** > dislocations for insufficient work hardening capability and semi‐coherent α/β interfaces for strain incompatibility. The strength–ductility trade‐off of duplex Ti alloys is further amplified by interstitial atoms‐poisoning effects (e.g., N and O). Here, by selecting N atoms with the strongest hardening ability in Ti alloys, a counterintuitive strategy is proposed that harnesses bifunctional N‐dislocation interactions in a model duplex Ti–Cr–Zr–Al alloy to construct a heterogeneous lamella structure, involving the elongated α_p_ grains decorated with N‐rich low‐angle grain boundaries (LAGBs) and densely coherent interstitial‐N α′‐nanotwinned martensites in β‐grains. This structural heterogeneity achieves extremely high yield/tensile strength of ≈1532/1869 MPa in our alloys, which in turn promotes the emission of massive < ***c*** + ***a*** > dislocations from N‐rich LAGBs and coherent interfaces through stress‐activated bow‐out and cross‐slip processes for relatively large ε_u_ ≈10.2%. This work thus opens an avenue, via bifunctional interstitial atom‐dislocation interactions, to construct a unique microstructure, toward ultrahigh strength and large ductility in interstitial‐strengthening Ti alloys.

## Graphical abstract

By engineering bifunctional nitrogen‐dislocation interactions, this work constructs a heterogeneous lamellar structure with N‐rich low‐angle boundaries and coherent nanotwinned martensites, enabling ultrahigh yield/tensile strength (1532/1869 MPa) and activating massive < ***c*** + ***a*** > dislocations via stress‐driven bow‐out/cross‐slip. This strategy delivers exceptional strength–ductility synergy (≈10.2% uniform strain) while overcoming the well‐known interstitial atoms‐embrittlement effect in conventional Ti alloys.

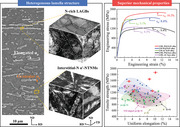

## 1 Introduction

Interstitial atoms (such as N and O) have garnered significant attention in metallurgy due to their exceptional strengthening capabilities for metallic materials—from conventional alloys to newly emerging high‐entropy alloys (HEAs)—primarily through creating greater lattice distortion than that caused by substitutional ones.^[^ ^1^, ^2^ ^]^ This interstitial doping has been successfully applied to steels^[^ ^3^, ^4^ ^]^ and HEAs.^[^ ^2^, ^5^, ^6^, ^7^, ^8^, ^9^ ^]^ Recent findings in HEAs, such as Fe_45.9_Mn_30.4_Co_10.5_Cr_10.6_N_2.6_ and Fe_49_Mn_30_Co_10_Cr_10_N_1_ (at.%) alloys, demonstrate novel interstitial engineering strategies, namely interstitial atoms increase stacking fault energy to switch deformation modes from transformation‐induced plasticity to twinning‐induced plasticity (TWIP),^[^ ^7^ ^]^ and mediate short‐range ordering to facilitate dislocation cross‐slip for strength–ductility synergy.^[^ ^2^, ^5^ ^]^ In contrast to conventional steels and interstitial‐strengthened HEAs, Ti alloys suffer severe ductility loss upon interstitial hardening – a phenomenon termed the interstitial embrittlement effect.^[^ ^1^, ^10^ ^]^ This strength–ductility trade‐off originates from the fact that interstitial atoms not only inducing planar slip to promote strain localization, but also increasing lattice friction stresses to inhibit TWIP, both of which lead to reduced ductility.^[^ ^11^, ^12^ ^]^ Recently proposed metallurgical designs in single‐phase hexagonal close‐packed (HCP) structure Ti alloys offer new insights into alleviating the interstitial embrittlement effect. Besides diluting the concentration of deleterious solutes (O and N) in grain boundaries via grain refinement,^[^ ^13^, ^14^ ^]^ another method is to interrupt the metastable shuffling mechanism of O atoms by strong repulsive of Al–O interactions to drive cross‐slip for ductility of Ti–Al alloys.^[^ ^13^ ^]^ Nevertheless, these single‐phase HCP‐Ti alloys universally suffer from inadequate yield strength (σ_0.2_ < 1200 MPa), even with the yield drop caused by the low work hardening rate (WHR, Θ).

Compared with single‐phase Ti alloys with low σ_0.2_, duplex (α+β) Ti alloys achieve high σ_0.2_ by tailoring their major constituent‐HCP α phase, for critical load‐bearing applications.^[^ ^15^, ^16^, ^17^, ^18^ ^]^ However, high‐strength duplex Ti alloys often confront low WHR and thus the limited uniform elongation (ε_u_), due to the following two factors. i) The HCP α‐phase often lacks < ***c*** + ***a*** > dislocations due to the excessive critical resolved shear stress (CRSS), rendering intrinsically limited ductility.^[^ ^19^, ^20^, ^21^, ^22^ ^]^ ii) The secondary α‐precipitate/β‐matrix semi‐coherent phase boundaries (PBs) lead to strain incompatibility and thus stress concentrations for reduced ductility.^[^ ^23^, ^24^ ^]^ More importantly, the strength–ductility trade‐off in duplex Ti alloys caused by the above two reasons is further amplified by interstitial atom‐poisoning effects.^[^ ^23^, ^25^ ^]^ This is because the strong interstitial atom‐dislocation interactions exacerbate the above two drawbacks, i.e., increasing the CRSS to suppress activation of < ***c*** + ***a*** > dislocations^[^ ^10^ ^]^ and strengthening the α‐phase to intensify strain incompatibility at α/β PBs.^[^ ^26^, ^27^ ^]^ So far, significant efforts have been made in duplex Ti alloys to alleviate the interstitial embrittlement effect.^[^ ^26^, ^27^, ^28^, ^29^, ^30^ ^]^ The well‐investigated systems are interstitial atom‐doped Ti‐6Al‐4V^[^ ^26^, ^29^, ^30^, ^31^ ^]^ and Ti–Fe^[^ ^1^ ^]^ alloys, but they still show quite limited ε_u_ ≤ 7%. For example, Ti‐6Al‐4V‐N alloys have the >1300 MPa‐class high tensile strength (σ_UTS_) and low ε_u_ < 5%,^[^ ^31^ ^]^ while 3D printed Ti–Fe–O alloys with O nano‐heteropartition possess high σ_0.2_ ≈ 1235 MPa but low ductility (ε_f_) ≈3%.^[^ ^1^ ^]^ Among the commonly incorporated interstitial atoms in Ti alloys, despite N has the strongest interstitial hardening capacity, it has been underexplored in duplex Ti alloys due to embrittlement concerns.^[^ ^15^, ^32^ ^]^ Therefore, how to utilize the exceptional strengthening capacity of interstitial N atoms with effective embrittlement mitigation remains a critical challenge in the development of high‐performance Ti alloys.

Here, we demonstrated that the adverse role of interstitial N atoms can be turned around so that they can elevate the ductility of duplex Ti alloys. We take a cue from the recent observations that the interstitial atom‐dislocation interactions not only promote the formation of strong substructures of solute segregation (e.g., low‐angle grain boundaries, LAGBs^[^ ^4^, ^33^, ^34^ ^]^) for pronounced strengthening, but also tend to segregate the dislocation core to mediate phase transformation.^[^ ^35^ ^]^ In this work, by selecting interstitial N atoms with the strongest hardening ability,^[^ ^10^, ^32^ ^]^ we developed a simple cyclic hot‐rolling and short‐time‐solution (HR&SS) processing to control N‐dislocation interactions at high temperatures for tailoring microstructures (i.e., α and β phases) in N‐doped Ti‐2.8Cr‐4.5Zr‐5.2Al alloys (Texts S1 and S2 for composition design and thermomechanical processing, Supporting Information). Specifically, for the primary α (α_p_) phase, the N‐dislocation interactions not only promote wavy slip to produce enough dislocations during hot‐rolling,^[^ ^16^, ^21^, ^32^ ^]^ but also prevent dislocation annihilation during short‐time solutioning,^[^ ^4^, ^33^ ^]^ which facilitates forming N‐rich LAGBs. For the high‐temperature β‐phase, this interaction distorts the planar stress fields of edge dislocations into the nonplanar stress fields, which promotes the formation of coherent and interstitial‐N α′‐nanotwinned martensites (α′‐NTNMs) during water quenching (WQ).^[^ ^17^, ^35^ ^]^ Thus, we obtain a heterogeneous lamella structure consisting of elongated α_p_ grains decorated with N‐rich LAGBs and β*_trans_* structure with interstitial‐N α′‐NTNMs that elevate the flow stress to reach the CRSS for activating < ***c*** + ***a*** > dislocations,^[^ ^34^, ^36^ ^]^ promoting N‐rich LAGBs and α′‐NTNM PBs as prolific dislocation sources, leading to wavy slip and the *c*‐axis deformation,^[^ ^33^, ^37^ ^]^ and interstitial‐N α′‐NTNMs coherent with the matrix to alleviate stress concentrations. This lamellar structure, designed by using bifunctional N‐dislocation interactions, lends N‐doped Ti‐Cr‐Zr‐Al duplex Ti alloys excellent mechanical properties with ultra‐high yield/tensile strength of ≈1532/1869 MPa and large uniform elongation of ε_u_ ≈10.2%, together with high work hardening capability as (σ_UTS –_ σ_0.2_) ≈337 MPa.

## 2 Results and Discussion

### 2.1 Heterogeneous Lamella Structure of the Nitrogen‐Doped Ti‐Cr‐Zr‐Al Alloys

**Figure 1:**
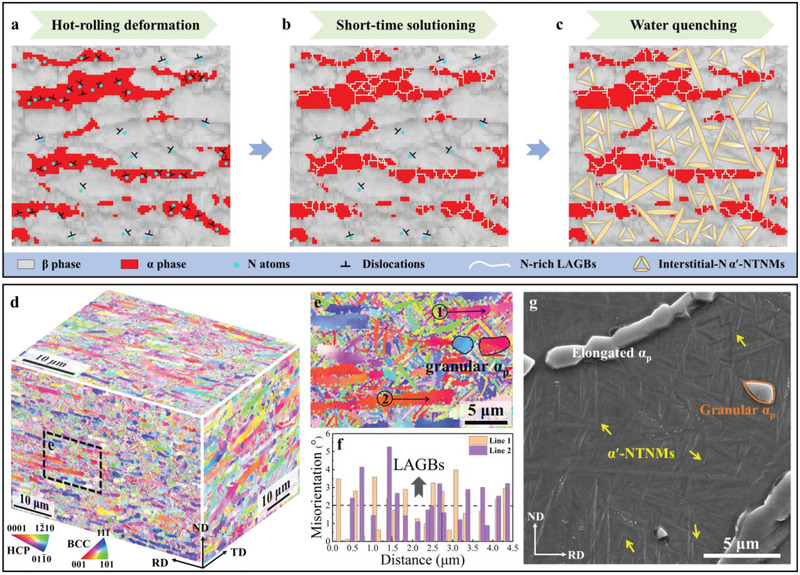
The heterogeneous lamella structure evolution of the LML‐WQ 0.4N alloy. a–c) Schematic illustration of the N‐dislocation interaction and microstructure evolution during HR&SS processing. d) A representative 3D electron‐backscatter‐diffraction (EBSD) inverse pole figure (IPF) showing the lamellar structure composed of α_p_ grains and β*_trans_* structure. ND is the normal direction, TD is the transverse direction, and RD is the rolling direction. e) The EBSD image taken from the area marked in (d), demonstrating the elongated and granular α_p_ grains and α′‐NTNMs. f) The corresponding point‐to‐point misorientation angle variation, along the black arrows in the elongated α_p_ grain in (e). g) The SEM image showing the α′‐NTNMs with triangular morphology in the β*_trans_* structure of the LML‐WQ 0.4N alloy.

Four batches of alloys were prepared, namely the base alloy, 0.3, 0.4, and 0.5 wt.% N‐doped Ti–Cr–Zr–Al alloys, to elucidate the N‐dislocation interactions on the microstructural features and mechanical properties. Based on the HR&SS processing (Route‐I in Figure S1, hereafter denoted as LML‐WQ N‐doped alloys, Supporting Information), interstitial N‐dislocation interactions occur in both α_p_ and β phases during hot‐rolling and short‐time solutioning (**Figure** **1**a,b). After WQ, N‐doped alloys all show similar heterogeneous lamella structures, in which elongated and granular α_p_ grains are formed (Figure S2, Supporting Information). Increasing N contents from 0.3 to 0.5 wt.%, the average grain size and the volume fraction of granular α_p_ increase from ≈1.4 to ≈2.3 µm and from ≈2.1% to ≈3.1% (Figure S3, Supporting Information), respectively. For the elongated α_p_, the short axis intercept increases from ≈1.2 to ≈2.5 µm, the long axis intercept increases from ≈8.2 to ≈11.5 µm, and the volume fraction increases from ≈22.5% to ≈25.3% (Figure S3, Supporting Information). Notably, the partially recrystallized elongated α_p_ grains are dominated by profuse LAGBs caused by N‐dislocation interactions that remained at room temperature (RT), while the granular α_p_ grains have completely recrystallized, as verified by the misorientation and transmission electron microscopy (TEM) analysis in LML‐WQ 0.4N alloys (Figure 1d–f; Figure S4, Supporting Information). Concurrently, metastable β‐phase transformed into interstitial‐N α′‐NTNMs with the triangular morphology by N‐dislocation interaction‐mediated diffusionless transformation (Figure 1c), forming a hierarchical structure (Figure 1g; Figures S2 and S5, Supporting Information). When the N content increases from 0.3 to 0.5 wt.% (Figure S5, Supporting Information), the average thickness and volume fraction of α′‐NTNMs increase from 28 ± 7 to ≈49 ± 9 nm and from ≈46.1% to ≈51.8% (Figure S5, Supporting Information), respectively. The above results show that N, as a strong α‐stabilizer, not only expands the α‐phase region but also increases the martensitic start temperature in Ti alloys, thus affecting the coarsening behavior of the α_p_ phase and α′‐NTNMs. This is consistent with the findings in N‐doped Ti‐6Al‐4V alloys.^[^ ^38^ ^]^

Given the optimum strength–ductility balance in the LML‐WQ 0.4N alloy (Figure S6, Supporting Information), we take it as an example for comprehensive microstructural and mechanical characterization. To confirm the critical roles of LAGBs and coherent PBs designed by bifunctional N‐dislocation interactions, we tailor the LML‐WQ 0.4N alloys by rapid heating to the rolling temperature (1020 °C, holding for 1 min) to diminish N‐dislocation interactions, followed by air cooling (AC) or WQ to RT (Route‐II in Figure S1, Supporting Information, hereafter denoted as LM‐AC 0.4N and LM‐WQ 0.4N alloys, respectively). Taking the LM‐AC 0.4N alloy as an example, this processing retains the elongated α_p_ grains but eliminates LAGBs, while the β‐matrix transforms into the α_s_ phase with an average thickness of 78 ± 9 nm (Figures S7 and S8, Supporting Information).

**Figure 2:**
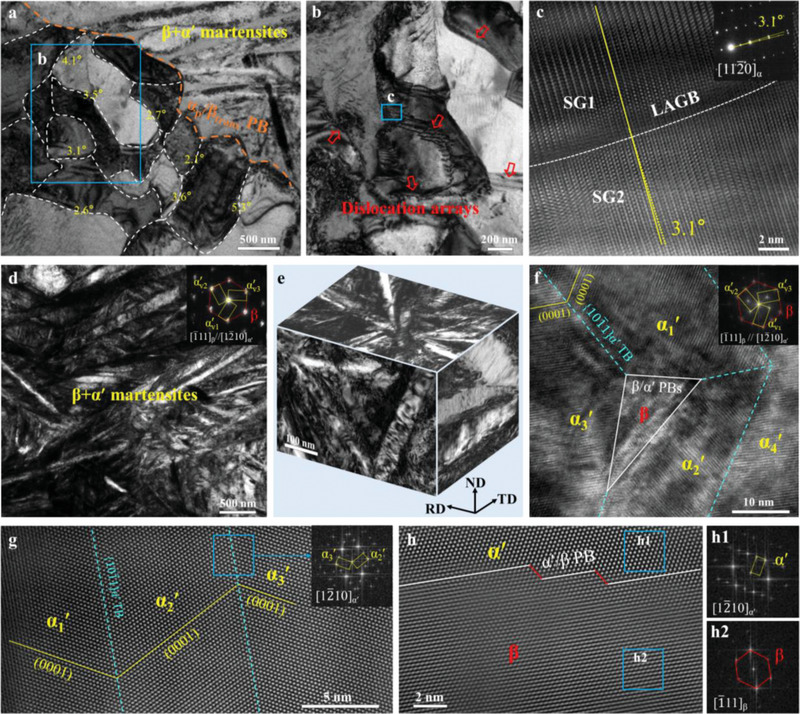
Microstructures of the LML‐WQ 0.4N alloy. a) Bright‐field (BF) TEM image showing elongated α_p_ grain and β*_trans_* structure, where the elongated α_p_ is composed of a large number of sub‐grains, as marked via the white dotted lines. The misorientation angle of some LAGBs is marked. b) BF‐TEM image taken from the area marked in (a), demonstrating that these LAGBs are composed of dislocation arrays. c) High resolution (HR) TEM and the corresponding Fast Fourier Transformation (FFT) images taken from the marked rectangular region in (b) showing a typical LAGB with a misorientation angle of 3.1°. d) BF‐TEM image showing α′‐NTNMs in the β*_trans_* structure. The inset is the corresponding SAED pattern, verifying twinned‐martensite variants. e) A 3D BF‐TEM image showing the pyramidal morphology of α′‐NTNMs in the β*_trans_* structure. f) The HR‐TEM image showing four α′‐martensites, and they are all separated by {10$\bar{1}$1}_α′_ TBs, as marked via cyan dotted lines. The PBs are marked by white lines. The inset is the corresponding FFT pattern. g) The high‐angle annular dark‐field scanning TEM (HAADF‐STEM) image showing two {10$\bar{1}$1}_α′_ CTBs. The inset is the FFT pattern of {10$\bar{1}$1}_α′_ α′‐twin variants. h) The HAADF‐STEM image showing the coherent α′/β phase interface. h1‐h2) FFT patterns correspond to areas outlined by solid squares in (h), respectively.

The bright field (BF) TEM image shows inside an elongated α_p_ grain, there are numerous sub‐grains (SGs, with an average size of ≈0.51 µm, **Figure** **2**a; Figure S9a, Supporting Information). Each SG boundary decorated with a high density of dislocations belongs to LAGBs having small misorientation angles ranging from 2.1° to 5.3° (Figure 2a). There are relatively fewer dislocations with curved morphologies inside the SG interior (Figure 2b; Figure S9b,c, Supporting Information). High‐resolution (HR) TEM observation conducted along the [11$\bar{2}$0] zone axis shows that one of the closely packed planes of SG1 is inclined by 3.1° relative to that of SG2 (Figure 2c). Moreover, the three orthogonal directions (including ND, TD, and RD) of LML‐WQ 0.4N alloys were characterized, confirming the formation of 3D LAGB networks within the elongated α_p_ grains, as shown in Figure S10 (Supporting Information). It appears that the LAGBs in elongated α_p_ grains are primarily formed via the mutual trapping and self‐organization of hot‐rolling‐induced dislocations.

For the β*_trans_* structure in LML‐WQ 0.4N alloys, the acicular α′‐nanomartensites with dislocation substructure prevailed in the β‐matrix after WQ (Figure 2d). Similarly, the 3D analysis reveals distinct morphological characteristics across different planes: a self‐accommodating plate group (SAPG)‐II with the hollow triangular morphology was observed on the TD plane,^[^ ^39^ ^]^ while V‐shaped martensite configurations were identified on both RD and ND planes (Figure 2e). This complex spatial arrangement differs significantly from conventional unidirectionally aligned lamellar structures, unveiling that the α′‐NTNM is a pyramidal morphology in 3D space.^[^ ^40^, ^41^ ^]^ The selected area electron (SAED) pattern provided in Figure 2d reveals that the β‐matrix and α′‐martensite obey the Burgers orientation relationship,^[^ ^16^, ^28^ ^]^ i.e., (0001)_α′_//(101)_β_ and [1$\bar{2}$10]_α′_//[$\bar{1}$11]_β_. The HR‐TEM image shows four α′‐martensites in the sample along [$\bar{1}$11]_β_, in which three of these martensites surround a β‐phase (Figure 2f). These four α′‐martensites belong to three variants, as verified by the fast‐Fourier transform (FFT) pattern (designated as α′_v1_, α′_v2_, and α′_v3_, respectively, inset in Figure 2f). The misorientation between any two of three α′‐variants is ≈60° (Figure 2f),^[^ ^17^ ^]^ forming hierarchical α′‐NTNMs structure with Type‐2 {10$\bar{1}$1}_α′_ twinning relationship (cyan dotted lines in Figure 2f,g; Table S1, Supporting Information). Moreover, these α′‐martensites share fully coherent α′/α′ TBs and α′/β PBs (Figure 2g,h2), with an average thickness *λ* of 35 ± 6 nm (Figure S5d, Supporting Information).

### 2.2 Origin of the LAGBs and α′‐NTNMs in Duplex LML‐WQ 0.4N Alloys

We next illustrate how the N‐dislocation interactions mediate the formation of N‐rich LAGBs and interstitial‐N α′‐NTNMs, respectively, in our N‐doped Ti alloys. A key finding here is that these LAGBs inside elongated α_p_ grains are mainly characterized by pyramidal < ***c*** + ***a*** > dislocations (Figure S9b,c, Supporting Information). The < ***c*** + ***a*** > dislocation is difficult to activate at RT in HCP alloys,^[^ ^19^, ^20^, ^21^ ^]^ but at high temperatures, the difference in CRSS between these slip systems is significantly reduced.^[^ ^16^ ^]^ On the other hand, high‐temperature deformation also alters the N‐dislocation interactions at RT by suppressing the “interstitial atoms‐induced shuffle mechanism,”^[^ ^14^ ^]^ which activates multiple slip systems and wavy slip (for dislocation multiplication), contributing to the formation of 3D dislocation networks (i.e., LAGBs). These activated dislocations in alloys attract solute segregation in such lattice‐distorted regions, especially interstitial N atoms.^[^ ^42^ ^]^ These N atoms can serve as pinning sites against dislocation motion and restrict the growth of sub‐grains via the Zener pinning effect.^[^ ^4^, ^33^ ^]^ Furthermore, abundant < ***c*** + ***a*** > dislocations have high slip resistance with low mobility and form dislocation locks with < ***a*** > dislocations to stabilize LAGBs,^[^ ^21^ ^]^ as verified by the LAGBs with < ***c+a*** > dislocations. Thus, the N atom‐dislocation interactions can effectively regulate dislocation wavy slip, accumulation, and recovery by our HR&SS processing to form N‐rich LAGBs.

**Figure 3:**
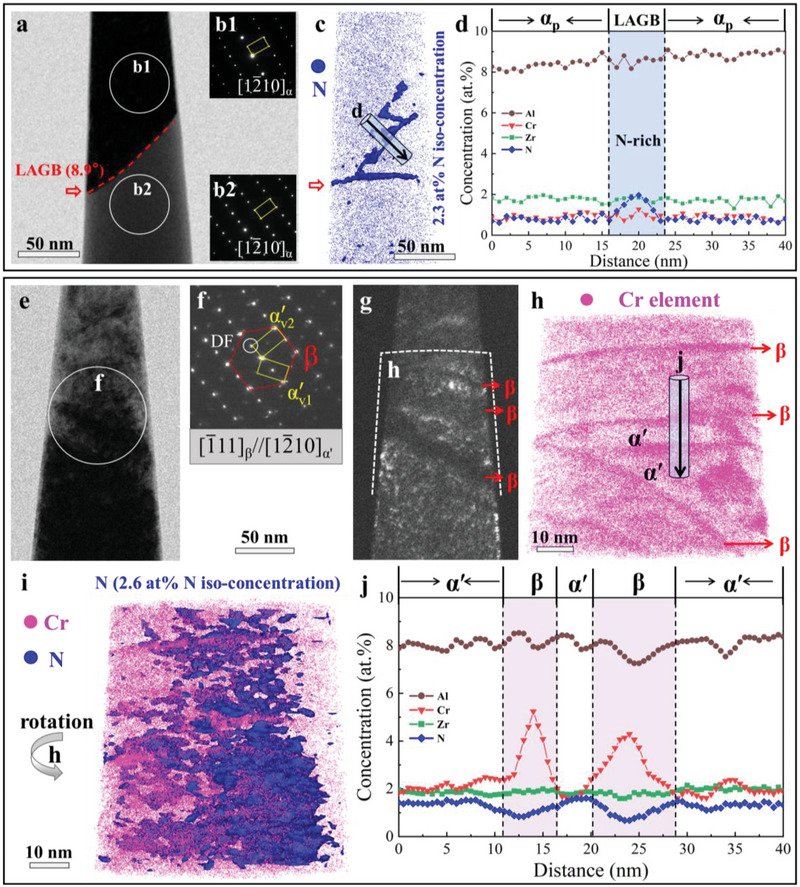
Elemental distributions in the LML‐WQ 0.4N alloy. a,b1‐b2) TEM images of an APT tip containing LAGBs in an elongated α_p_ grain. c) The APT 3D reconstructions with 2.3 at.% N iso‐concentration surfaces, showing a N‐rich LAGB. d) The 1D concentration profiles across the LAGB along the cylinder. e–g) TEM images of an APT tip containing α′‐NTNMs. h) The APT 3D reconstructions with the Cr element. i) Atom map of Cr, and corresponding 2.6 at.% N iso‐concentration surface. j) The 1D concentration profiles across the β‐matrix and α′‐NTNMs along the cylinder.

To unveil the features of impurity segregation on LAGBs, local chemical distribution was examined by correlative TEM and 3D‐APT. Here, TEM was first performed to locate the LAGBs in elongated α_p_ grains, and then the APT specimen containing LAGBs was prepared for detailed analyses (**Figure** **3**a). The SAED analysis for the APT sample shows that the orientation difference between these two HCP α_p_ grains is ≈8.9° (Figure 3b1,b2). Thus, the TEM results are direct evidence of the presence of a LAGB (red dotted line in Figure 3a) in the APT specimen, see Figure 3c. From the 3D reconstruction with the N element at 2.3 at.% N iso‐concentration surface, one can observe a much higher N concentration at the LAGB relative to the α_p_ grain interiors (Figure 3c). The 1D composition profile across the LAGB revealed that the N concentration is ≈1.89 at.% at the LAGB (Figure 3d), which is much higher than that inside the α_p_ grain (≈0.61 at.%). Our APT result is similar to previously reported findings that the N or O segregation emerges at crystal defects (such as twin boundaries^[^ ^32^ ^]^ and grain boundaries^[^ ^36^ ^]^) in HCP‐Ti.

For the α′‐NTNMs structure, given that the elastic interaction between α′‐martensites and pre‐existing dislocations dominates the variant selection of the martensitic transformation,^[^ ^17^, ^43^ ^]^ we characterize the dislocation types before phase transformation. Different from the elongated α_p_ grains decorated with LAGBs, individual edge dislocations are prevalently detected in the β‐matrix before martensitic transformation (Figure S11, Supporting Information). This is because, compared with the HCP α‐phase, the BCC β‐phase is more prone to massive annihilation of dislocations due to the higher growth kinetics.^[^ ^44^ ^]^ However, the planar stress field of an edge dislocation generally promotes the formation of one variant but inhibits the other two equivalent variants (as verified by our molecular dynamics (MD) simulations, see Figure S12a–d, Supporting Information),^[^ ^17^, ^43^ ^]^ namely, it is difficult for the edge dislocations to create such α′‐NTNMs. In fact, due to the strong interactions between the edge dislocations and interstitial N atoms, the N atomic atmosphere should be formed around the dislocation core.^[^ ^42^ ^]^ In this case, the N atoms would change the edge dislocation core stress field from planar to nonplanar, facilitating multiple variants of nucleation simultaneously along N‐rich edge dislocations, as verified by our MD simulations (Figure S12e–h, Supporting Information). Meanwhile, since the {10$\bar{1}$1}_α′_ coherent TB is a low‐energy interface; these variants could drive to adjust locally to generate {10$\bar{1}$1}_α′_ coherent TBs by lattice arrangement when they impinge together,^[^ ^17^ ^]^ i.e., forming the α′‐NTNMs.

To confirm α′‐NTNMs are mediated by N‐rich dislocations, we also conducted TEM‐APT analysis to characterize the composition of α′‐NTNMs, in particular the distribution of N atoms. The TEM images in Figure 3e–g clearly show the α′‐NTNMs in the APT specimen. The APT result showed that some Cr‐rich regions correspond to β lamellae in the TEM image (red arrows in Figure 3g,h), with the Cr concentration spanning from ≈4 at.% to ≈5 at.%. This 3D rendition with the Cr and 2.6 at.% N iso‐concentration surface maps also confirm the pyramidal arrangement of N‐rich α′‐martensites (Cr‐depleted in Figure 3h,i). 1D concentration profiles in Figure 3j show that the N atoms are enriched in α′‐martensites with the peak N‐content ≈1.4 at.%, which supports the MD simulations that the N‐rich edge dislocations trigger the α′‐NTNMs.

### 2.3 Mechanical Properties of the N‐Doped Ti–Cr–Zr–Al Alloys

**Figure 4:**
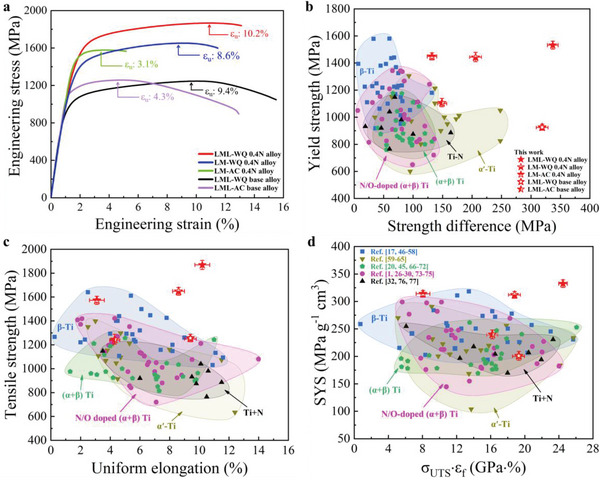
Mechanical properties of the base and 0.4N alloys with different microstructures. a) Tensile stress–strain curves for the LML‐WQ and AC base alloys, LML‐WQ 0.4N, LM‐AC, and WQ 0.4N alloys. b) The yield strength σ_0.2_ vs strength difference (σ_UTS_ – σ_0.2_), showing the marked strain hardening ability of the LML‐WQ 0.4N alloy. c,d) Maps of σ_UTS_ vs ε_u_ and specific yield strength (SYS) vs the product of σ_UTS_ and ε_f_ of our base and N‐doped Ti–Cr–Zr–Al alloys compared with those of previously reported including β‐Ti alloys,^[^ ^17^, ^46^, ^47^, ^48^, ^49^, ^50^, ^51^, ^52^, ^53^, ^54^, ^55^, ^56^, ^57^, ^58^ ^]^ martensite α′ Ti alloys,^[^ ^59^, ^60^, ^61^, ^62^, ^63^, ^64^, ^65^ ^]^ (α+β) Ti alloys,^[^ ^20^, ^45^, ^66^, ^67^, ^68^, ^69^, ^70^, ^71^, ^72^ ^]^ O/N‐doped (α+β) Ti alloys^[^ ^1^, ^26^, ^27^, ^28^, ^29^, ^30^, ^73^, ^74^, ^75^ ^]^ and N‐doped HCP‐Ti.^[^ ^32^, ^76^, ^77^ ^]^ Error bars in (b), (c), and (d) indicate the standard deviations for five tests. The detailed mechanical properties of base and N‐doped alloys can be found in Tables S2 and S3 (Supporting Information).

We here demonstrate that the LML‐WQ 0.4N Ti alloys designed by interstitial N‐dislocation interactions stand out in mechanical properties. **Figure** **4**a presents the engineering stress–strain curves of the base and 0.4N alloys with different microstructures. The base alloys undergoing WQ and AC conditions show low yield strength σ_0.2_ (in the range of 900–1100 MPa), while due to the lack of < ***c*** + ***a*** > dislocations and the strain incompatibility of semi‐coherent α_s_/β PBs, the latter shows limited uniform elongation (ε_u_ ≈4.3%) and low strain hardening capability, which is a typical stress–strain response of duplex Ti alloys.^[^ ^45^ ^]^ Compared with base alloys, these 0.4N alloys manifest notably increased strength. However, when the N‐dislocation interactions are eliminated by the rapid heating process (Route‐II in Figure S1, Supporting Information), the tensile ductility of both AC and WQ N‐doped alloys is lower than that of the base alloys. In particular, the LM‐AC 0.4N alloys (without LAGBs and coherent PBs) confirm the conventional wisdom of the interstitial atoms‐poisoning effects in duplex Ti alloys,^[^ ^26^, ^31^ ^]^ i.e., the uniform elongation ε_u_ sharply dropped to ≈3.2% and the total elongation ε_f_ to ≈5.1%. In contrast, the LML‐WQ 0.4N alloys (with LAGBs and coherent PBs) exhibit the highest σ_0.2_ of ≈1532 ± 23 MPa and σ_UTS_ of ≈1869 ± 18 MPa, associated with significant strain hardening capability thus large ε_u_ ≈10.2%, which is higher than that of the WQ base alloy (ε_u_ ≈9.7%). The Θ as a function of strains for the LML‐WQ 0.4N alloy in Figure S13 (Supporting Information) indicates that this microstructural feature guarantees a high strain‐hardening rate over the entire uniform deformation range (Θ > 2 GPa before necking), which is in sharp contrast to the sharply reduced Θ upon straining in LM‐AC 0.4N samples. Our alloy design strategy not only successfully breaks the long‐standing strength–ductility trade‐off prevailing in Ti alloys, but also overcomes the well‐known interstitial atoms‐embrittlement effect in conventional Ti alloys.

We summarize in Figure 4b the strength difference (σ_UTS_ − σ_0.2_) vs the σ_0.2_ for our designed and previously reported high‐performance Ti alloys, including β‐Ti alloys,^[^ ^17^, ^46^, ^47^, ^48^, ^49^, ^50^, ^51^, ^52^, ^53^, ^54^, ^55^, ^56^, ^57^, ^58^ ^]^ martensite α′ Ti alloys,^[^ ^59^, ^60^, ^61^, ^62^, ^63^, ^64^, ^65^ ^]^ (α+β) Ti alloys,^[^ ^20^, ^45^, ^66^, ^67^, ^68^, ^69^, ^70^, ^71^, ^72^ ^]^ O/N‐doped (α+β) Ti alloys^[^ ^1^, ^26^, ^27^, ^28^, ^29^, ^30^, ^73^, ^74^, ^75^ ^]^ and N‐doped HCP‐Ti.^[^ ^32^, ^76^, ^77^ ^]^ Our LML‐WQ 0.4N alloys exhibit a yield strength comparable with some of the strongest existing high‐strength β‐Ti alloys (σ_0.2_ >1500 MPa)—but with a strength difference (σ_UTS_ − σ_0.2_) ≈337 MPa that is almost a factor of seven greater. It indicates a large safety margin of the LML‐WQ 0.4N alloy, which is of great importance for engineering materials to prevent accidents from a sudden rupture in service. This high work hardening ability enhances the uniform elongation of alloys. As we show in Figure 4c an Ashby map of the σ_UTS_ vs ε_u_, this exceptional strength–ductility combination sets our LML‐WQ 0.4N Ti alloys apart from all reported Ti alloys. The combination of specific yield strength (SYS, ≈332 MPa·g^−1^·cm^3^) and the production (~24.5 GPa %) of σ_UTS_ × ε_f_ of the LML‐0.4N Ti alloy causes it to outperform high‐strength β‐Ti alloys, which are particularly renowned for excellent specific strength for lightweight applications,^[^ ^78^ ^]^ see Figure 4d.

### 2.4 Underlying Mechanisms for Ultrahigh Strength

**Figure 5:**
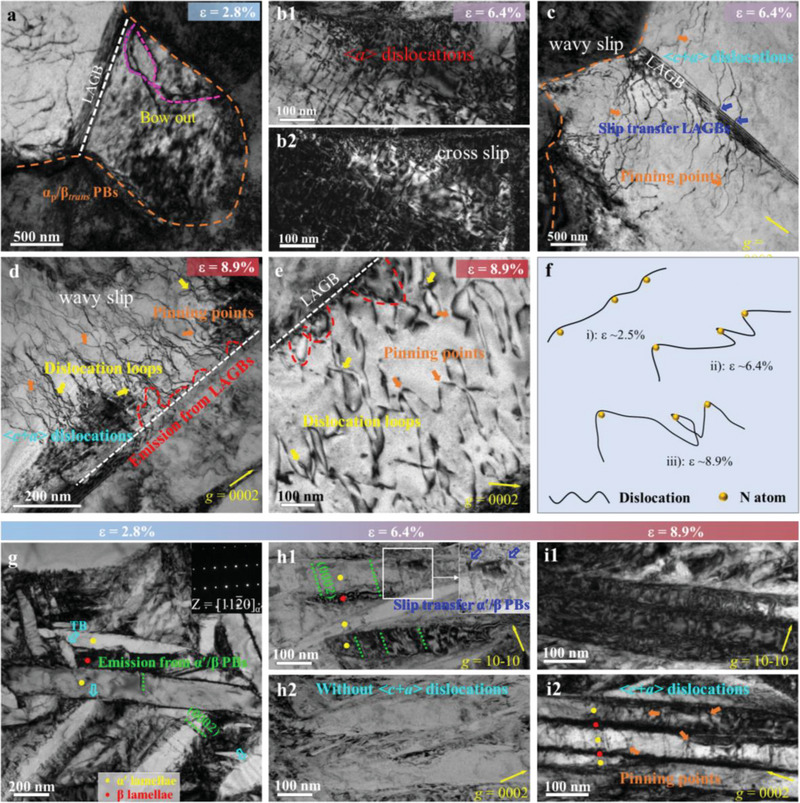
The deformed substructure of elongated α_p_ grain and β*_trans_* structure for the LML‐WQ 0.4N alloy with different interrupted strains. a) The TEM image of the deformation substructures of the elongated α_p_ grain at the tensile strain of ≈2.8%. b1‐b2,c) tensile strain ≈6.4%. d,e) tensile strain ≈8.9%. a) The BF‐TEM image shows dislocations first emitted from the α_p_/β*_trans_* interface and present a curved shape, marked by pink dotted lines. b1‐b2) BF‐TEM and corresponding weak beam dark field images in the α_p_ grain, showing the cross slip of < ***a*** > dislocation. c) BF‐TEM image demonstrates that some < ***c*** + ***a*** > dislocations with curved shapes were activated inside the α_p_ grains. Dislocations crossed the LAGB into the adjacent grain, highlighted by the blue arrows. Some < ***c*** + ***a*** > dislocations with wavy shapes indicate a strong O‐pinning effect, as marked by orange arrows. d,e) BF‐TEM image shows massive < ***c*** + ***a*** > dislocations with bow‐out shapes near LAGB inside the elongated α_p_ grains, indicating that LAGBs in LML‐WQ 0.4N sample appear to be an efficient source for < ***c*** + ***a*** > dislocations. Dislocation loops formed by N atom‐dislocation interactions are observed, as marked by yellow arrows. f) Schematic of the evolution of N‐dislocation interaction to form a typical dislocation loop. g, h1‐h2 and i1‐i2) The BF‐TEM images and two‐beam condition analysis of the β*_trans_* structure at tensile strains of ≈2.8% (g), ≈6.4% (h1‐h2) and ≈8.9% (i1‐i2). g) BF‐TEM image showing the basal < ***a*** > dislocation emission event (green dotted lines). TBs were highlighted by cyan arrows. h1‐h2) Two‐beam condition analysis in α′‐nanotwinned nanomartensites shows that < ***a*** > dislocations dominate in the α′‐martensites. Dislocation transmission across α′/β PBs, highlighted by blue arrows. i1‐i2) Two‐beam condition analysis shows massive < ***c+a*** > dislocations with way‐slip inside the α′‐martensites, indicating a strong O pinning effect, as marked by orange arrows. BCC (indicated by the red dot) and HCP (indicated by the yellow dot) nanolamellae.

The resulting excellent mechanical properties of the present LML‐WQ 0.4N alloy originate from the unique microstructure features designed by harnessing bifunctional N‐dislocation interactions. We now discuss the underlying mechanisms for the ultrahigh yield strength observed. First, LAGBs can be easily penetrated by dislocations due to small differences in slip vectors between adjacent grains, thereby showing weak resistance to dislocations in traditional wisdom. However, the N‐rich LAGBs maintain their shape/size and show high resistance to incoming dislocations during the entire plastic deformation, see **Figure** **5**a–e. In other words, unlike traditional LAGBs, the strengthening ability of LAGBs with N‐segregation has been demonstrated to be comparable with that of high‐angle GBs, as observed in steels^[^ ^33^ ^]^ and HEAs.^[^ ^37^ ^]^ The 3D LAGBs effectively subdivide elongated α_p_ grains (Figure S10, Supporting Information), providing significant strengthening through grain refinement. Thus, the elongated α_p_ grains decorated with N‐rich LAGBs in the duplex Ti alloy achieve high strength. Second, the relation between the yield strength and the interstitial‐N α′‐NTNMs with the average thickness *λ*_α′_ of ≈37 nm follows the Hall–Petch relationship (for both coherent α′/β and α′/α′ interface strengthening).^[^ ^63^, ^79^ ^]^ Especially, the pyramidal α′‐NTNMs in 3D space with Type‐II coherent twin boundary exhibit higher phase boundary density compared to other α′/α′ interfaces.^[^ ^79^ ^]^ Hence, the effective grain refinement achieved by the formation of ultra‐dense α′‐NTNMs structure significantly reduces the mean free path of dislocations. Finally, the present LML‐WQ 0.4N alloy shows a heterogeneous lamella microstructure, i.e., soft α_p_ and hard β*_trans_*. During tensile deformation, the soft α_p_ grains underwent higher plastic strains to generate geometrically necessary dislocations (GNDs) near α_p_/β*_trans_* PBs to accommodate heterogeneous deformation (Figure 5a). This phenomenon is well‐known to render the hetero‐deformation‐induced (HDI) strengthening (Figure S14, Supporting Information). Based on the rule‐of‐mixture model, we calculate the yield strength ≈1524 MPa of the present LML‐WQ 0.4N alloy, in agreement with the experimentally measured value ≈1532 MPa (Figure S15, Supporting Information). It is found that the strengthening contributions from solid–solution strengthening (The phase compositions of LML‐WQ 0.4N alloys measured from APT analysis, see Table S4, Supporting Information), HDI strengthening, and hetero‐phase interface strengthening are ≈590, ≈662 and ≈272 MPa, respectively, (the detailed calculation process refer to Text S3 and Table S5, Supporting Information).

### 2.5 Underlying Mechanisms for Large Ductility

Next, we reveal the underlying mechanisms responsible for the large uniform elongation in the LML‐WQ 0.4N alloy through TEM observations. At a low strain of ≈2.8%, the dislocations were first activated at α_p_/β*_trans_* PBs due to heterogeneous deformation and are blocked by the N‐rich LAGBs inside elongated α_p_ grains (Figure 5a), generating Hall–Petch strengthening. Note that these activated dislocations exhibit pronounced curvature, indicative of a localized N pinning effect.^[^ ^6^ ^]^ In the β*_trans_* structure, α′/β PBs serve as dislocation sources to emit dislocations for enhanced ductility (green lines in Figure 5g). Different from the observed dislocations at the α′/β PBs, the lower energy α′/α′ TB is more stable and the slip system is more difficult to activate,^[^ ^80^ ^]^ as confirmed by the smooth and clean TBs (cyan arrows and corresponding atomic‐scale TBs observation, see Figure 5g; Figure S16a,b, Supporting Information).

When the strain increases to ≈6.4%, the pre‐existing N‐rich LAGBs are still clearly visible in the elongated α_p_ grain (Figure S17a,b, Supporting Information). We detected the typical tangle of < ***a*** > dislocations commonly found in previously studied Ti and its alloys since it has the lowest CRSS (Figure 5b1‐b2; Figure S17c1–c2, Supporting Information).^[^ ^16^ ^]^ When imaged with ***g*** = (0002), it is seen that some < ***c*** + ***a*** > dislocations were emitted from the α_p_/β*_trans_* PBs (Figure 5c). Interestingly, although many < ***c*** + ***a*** > dislocations are obstructed by N‐rich LAGBs, several dislocations still overcame the impediment of N‐rich LAGBs and glided into the neighboring SGs (blue arrows in Figure 5c). This result indicates that these N‐rich LAGBs act as stable and moderate barriers to hinder dislocation motion for strength at low strains, while guaranteeing ductility by dislocation transmission to release stress concentration at medium strains. More importantly, these activated < ***a*** > and < ***c*** + ***a*** > dislocations show evident wavy/cross slip behavior (Figure 5b1‐b2,; Figure S17, Supporting Information), which is far different from the conventional perspective that interstitial atom‐induced planar slip.^[^ ^13^ ^]^ This can be attributed to the following two aspects. i) Due to the strong N pinning effect, typical bow‐out dislocation features were observed (orange arrows in Figure 5c). ii) The limited room of SGs (with the average size ≈0.51 µm, Figure S9a, Supporting Information) in elongated α_p_ grain constraints the planar slip. Moreover, the 3D N‐rich LAGBs also disturb the propagation of planar slip to promote the development of wavy slip.^[^ ^36^ ^]^ By contrast, there is an obvious planar slip in the LM‐AC 0.4N alloy (without LAGBs, Figure S18, Supporting Information). Therefore, our N‐dislocation interactions and N‐rich LAGBs effectively eliminate the planar slip caused by the “interstitial atom‐induced shuffle mechanism.”^[^ ^14^ ^]^ For the β*_trans_* structure, only long and straight < ***a*** > dislocations were observed in α′‐martensites (Figure 5h1‐h2). Due to the good slip continuity across the α′/β interfaces, these basal < ***a*** > dislocations can easily transmit across them to alleviate stress concentrations (blue arrows in Figure 5h1). Compared with α′/β coherent PBs, the α′/α′ coherent TBs have a stronger resistance to dislocation slip, as confirmed by the accumulation of dislocations at α′/α′ TBs (Figure S16c,d, Supporting Information). Therefore, the TBs in α′‐NTNMs structure interact more effectively with dislocations, which is favorable for higher work hardening capability.

With further increasing the strain to ≈8.9%, massive < ***c*** + ***a*** > dislocations with wavy slip are activated and homogeneously distributed inside the entire elongated α_p_ grains and α′‐NTNMs (Figure 5d‐e, i1‐i2; Figure S19, Supporting Information), far different from previous finding of absence of < ***c*** + ***a*** > dislocations in Ti alloys.^[^ ^20^, ^21^ ^]^ The lack of < ***c*** + ***a*** > dislocations is an intrinsic limitation in Ti and its alloys due to the required high CRSS. In particular, the interstitial atoms further increase the activated CRSS of < ***c*** + ***a*** > dislocations and planar slip,^[^ ^14^, ^26^, ^81^ ^]^ resulting in the interstitial‐embrittlement effect. For example, in the reference LM‐AC 0.4N sample with limited ductility (brittle‐like fracture in Figure S20, Supporting Information), we observed a limited number of < ***c*** + ***a*** > dislocations in planar slip inside the α phase (Figure S18, Supporting Information). The activation of massive < ***c*** + ***a*** > dislocations in the LML‐WQ 0.4N alloy is attributed to the heterogeneous lamella structure designed by bifunctional N‐dislocation interactions. i) The combination of multiple strengthening mechanisms to reach the true stress up to 2 GPa (Figure S13, Supporting Information), overwhelming the required high CRSS of < ***c*** + ***a*** > dislocations. ii) The 3D N‐rich LAGBs act as prolific dislocation sources, where stress‐driven interactions between mobile dislocations and solute‐decorated LAGBs trigger self‐multiplication via bow‐out and cross‐slip mechanisms.^[^ ^32^, ^36^ ^]^ iii) Randomly oriented 3D boundaries in α′‐NTNMs facilitate multi‐slip system interactions, unlike the localized slip in unidirectional lamellar structures,^[^ ^82^ ^]^ thereby promoting < ***c*** + ***a*** > dislocation activation. At large strains, the wavy slip from N‐dislocation interactions evolves into dislocation loops (see yellow arrows in Figure 5d,e). The schematic diagram of Figure 5f shows the influence of N‐dislocation interaction on the evolution behavior of dislocation slip. First, N anchoring induces dislocation line curvature. Second, stress further drives the dislocation line to exhibit a significant bending pattern. Finally, the mobile segment of the dislocation rotates along with the N pinning points to form a dislocation loop through cross‐slip. Hence, the N‐dislocation interaction promotes the wavy slip and cross‐slip by mediating the formation of dislocation loops in elongated α_p_ grains, improving the WHR of the alloy. Similarly, the N‐dislocation interactions change from the straight dislocation lines at medium strains (Figure 5h1‐i2) in α′‐NTNMs to wavy slip at high strains (orange arrows in Figure 5i1‐i2), further confirming the N pinning effect. Moreover, the α′/α′ coherent TBs still have a stronger resistance to dislocation slip, and thus can accommodate more dislocations at the TBs to accommodate plastic deformation (Figure S16e,f, Supporting Information). As a result, our heterogeneously lamellar structure designed by N‐dislocation interactions provides a self‐hardening mechanism for high strength and enhanced ductility.

## 3 Conclusion

In summary, we proposed a novel strategy that harnesses bifunctional N‐dislocation interactions to tailor the microstructure of duplex Ti alloys. Utilizing such a strategy, the ultra‐strong and ductile Ti–Cr–Zr–Al alloys can be achieved by a heterogeneous lamella structure that consists of elongated α_p_ grains decorated with N‐rich LAGBs and β*_trans_* structure with coherent and interstitial‐N α′‐NTNMs. This structure, combining LAGBs and coherent α′‐NTNMs interfaces with the moderate barriers, achieves extremely high flow stress in our alloys, which in turn promotes the emission of massive < ***c+a*** > dislocations from these interfaces for high strength and enhanced ductility. This work offers a novel strategy, via bifunctional interstitial atom‐dislocation interactions, to construct a unique microstructure, toward ultrahigh strength and large ductility in interstitial‐strengthening Ti alloys.

## 4 Experimental Section

### Materials Fabrication

The Ti‐2.8Cr‐4.5Zr‐5.2Al‐*x*N (*x* = 0, 0.3, 0.4 and 0.5 in wt.%) alloys were fabricated by vacuum consumable arc melting furnace, using the mixture of high purity sponge of Ti (purity > 99.9 wt.%), metal block of Cr (purity > 99.9 wt.%) and high purity billet of Zr (purity > 99.9 wt.%) and Al (purity > 99.9 wt.%), and TiN power (99.9 wt.%), respectively. The total weight of raw materials for each alloy sample was ≈80 g. For the 0.3, 0.4, and 0.5 N alloys, the addition amounts of TiN powder were 0.928, 1.237, and 1.856 g, respectively. The TiN powder was wrapped in Al foil to prevent the arc from blowing it away. The raw materials were remelted at least five times to guarantee chemical homogeneity in a high‐purity argon atmosphere. The raw materials were remelted at least five times to guarantee chemical homogeneity in a high‐purity argon atmosphere. The ingot was cast into a 14 × 10 × 55 mm^3^ water‐cooled copper mold. The β‐transus temperatures (T*_β_*) of base, 0.3N, 0.4N and 0.5N alloys determined by the metallographic method 950 ± 5, 1000 ± 5, 1040 ± 5, and 1080 ± 5°C, respectively. Homogenization was performed at 1200 °C for 60 min in an Ar atmosphere, followed by water quenching, and then cut into rectangular slabs with a thickness of 10 mm. To obtain similar microstructures, the homogenized specimens were processed and heat‐treated in the same way based on the T*_β_* temperature, i.e., cyclic hot‐rolling and short‐time‐solution (HR&SS) processing to fabricate a heterogeneous lamella structure Ti alloy, see Route‐I in Figure S1 (Supporting Information). For example, Ti‐2.8Cr‐4.5Zr‐5.2Al‐0.4N alloys were hot‐rolled at ≈1020 °C (≈20 °C below the T*_β_*). After each rolling pass with a reduction in thickness of 10%, the specimen was heated to the furnace temperature ≈1020 °C for ≈1 min, and until the total thickness reduction of the specimen reached 80%. Finally, these rolled samples underwent solution treatment at ≈1020 °C for 1 min. Similarly, other alloys with different N content are also thermomechanical processing based on T*_β_* temperature, the detailed processing procedure can be found in Text S1 and Figure S1 (Supporting Information).

### Microstructural Characterization

To study the microstructures at the nanoscale, a transmission electron microscope (TEM, JEM‐200) operated at 200 kV was employed. TEM foils were prepared by following standard electro‐polishing techniques for Ti alloys. Needle‐shaped specimens required for APT were fabricated by lift‐outs and annular milled in an FEI Scios focused ion beam/scanning electron microscope (FIB/SEM). The APT experiments were performed on a CAMECA local electrode atom probe (LEAP 4000XSi) under a high vacuum (Pa) at 20 K. Then, using the Integrated Visualization & Analysis Software (IVAS) version 3.6.8 for the 3D reconstructions and compositional analyses of the APT data of the specimens. The elemental distributions of different phases were measured by using the APT measurements. The chemical compositions of base and 0.4N alloys were measured by using inductively coupled plasma atomic emission spectroscopy (ICP‐AES) for metallic elements and LECO ONH836 Elemental Analyzer for non‐metallic elements, as listed in Table S6 (Supporting Information). A scanning electron microscope (SEM, JSM‐7001F) was used to characterize the microstructure of the preset alloys before and after the tensile tests. The thickness of the martensite was measured using image‐pro software.

### Measurements of Mechanical Properties

Before mechanical testing, all samples were mechanically ground at least 0.2 mm, followed by polishing and etching so as to inspect the deformation surface morphology and eliminate the possible influence of the oxidation effects during hot rolling. Dog‐bone specimens with gauge dimensions of 15 × 3.2 × 1.6 mm^3^ were cut parallel to the rolling direction (RD) for quasi‐static uniaxial tensile tests. The experiment was performed on an Instron 5969 universal testing machine at room temperature, with an initial strain rate of 1 × 10^−3^ s^−1^. All tests were repeated at least five times to ensure data reproducibility. The condition for the loading–unloading–reloading (LUR) test was the same as that of the monotonic tensile test. In each cycle, the specimen was first stretched to a designated strain at a rate of 1 × 10^−3^ s^−1^, and then unloaded by the stress‐control mode to 20 N at a rate of 200 N·min^−1^, followed by reloading at a rate of 1 × 10^−3^ s^−1^ again to the same applied stress before the next unloading.

### Molecular Dynamics Simulation

A modified embedded‐atom method (MEAM) potential developed by Lee et al. was employed to describe the interatomic interaction in the Ti–N system.^[^ ^83^ ^]^ To reveal the role of dislocation in the formation of quenched microstructure, a ½<111>_β_ edge dislocation dipole (Figure S12, Supporting Information) was created in the β phase via Volterra displacement field. The dipole had a separating distance of 19 nm. The simulation box was oriented as *x*‐[111], *y*‐[11$\bar{2}$], *z*‐[1$\bar{1}$0] for edge dislocation. The dimension of the model had the size of 17.2 nm × 37.2 nm × 19.2 nm for the edge dislocation. Periodic boundary conditions were applied in all directions. A certain number of N atoms was then introduced into the octahedral interstitial sites around the dislocation core. Before the loading was applied, the model was relaxed at 1200 K for 10 ps under an isothermal–isobaric thermostat. A hydrostatic tensile strain was applied to the sample at a strain rate of 10^9^ s^−1^ and a cooling rate of 1.8 × 10^13^ K s^−1^. All the calculations were carried out using LAMMPS code.^[^ ^84^ ^]^ The atomic configurations were displayed by OVITO.^[^ ^85^ ^]^

## Conflict of Interest

The authors declare no conflict of interest.

## Author Contributions

J.Y.Z. and J.S. conceived the research. C.L.Z. prepared the alloys and carried out most of the microscopy and all the mechanical property testing. X.Z.L. and S.Z.L. did the MD simulations. C.L.Z. and J.Y.Z. analyzed and interpreted the data, and wrote the manuscript with input from G.L. All co‐authors contributed to the data analysis and discussion.

## Supporting information



Supporting Information

## Data Availability

The authors declare that all data supporting the findings of this study are available within the paper and Supplementary Information files.

## References

[1] T. Song , Z. Chen , X. Cui , S. Lu , H. Chen , H. Wang , T. Dong , B. Qin , K. C. Chan , M. Brandt , X. Liao , S. P. Ringer , M. Qian , Nature 2023, 618, 63. 37259002 10.1038/s41586-023-05952-6 PMC10232360

[2] L. Sun , Z. He , N. Jia , Y. Guo , S. Jiang , Y. Yang , Y. Liu , X. Guan , Y. Shen , H.‐L. Yan , P. K. Liaw , Sci. Adv. 2024, 10, adq6398. 10.1126/sciadv.adq6398 PMC11606435 39612326

[3] Y. Chen , H. Zhu , P. Zhang , Z. Wang , M. Wang , G. Sha , H. Lin , J. Ma , Z. Zhang , Y. Song , P. Zheng , L. Zhou , S. Li , H. Liu , L. Shen , C. Qiu , 2023, Acta Mater. 250, 118868.

[4] B. Gao , Q. Lai , Y. Cao , R. Hu , L. Xiao , Z. Pan , N. Liang , Y. Li , G. Sha , M. Liu , H. Zhou , X. Wu , Y. Zhu , Sci. Adv. 2020, 6, aba8169. 10.1126/sciadv.aba8169 PMC7531883 32967821

[5] Z. He , Y. Guo , L. Sun , H.‐L. Yan , X. Guan , S. Jiang , Y. Shen , W. Yin , X. Zhao , Z. Li , N. Jia , Acta Mater. 2023, 243, 118495.

[6] M. Y. He , Y. F. Shen , N. Jia , P. K. Liaw , Appl. Mater. Today 2021, 25, 101162.

[7] Z. He , N. Jia , H. Yan , Y. Shen , M. Zhu , X. Guan , X. Zhao , S. Jin , G. Sha , Y. Zhu , C. T. Liu , Int. J. Plast. 2021, 139, 102965.

[8] Z. He , N. Jia , H. Wang , H. Yan , Y. Shen , J. Mater. Sci. Technol. 2021, 86, 158.

[9] Z. Lei , X. Liu , Y. Wu , H. Wang , S. Jiang , S. Wang , X. Hui , Y. Wu , B. Gault , P. Kontis , D. Raabe , L. Gu , Q. Zhang , H. Chen , H. Wang , J. Liu , K. An , Q. Zeng , T. G. Nieh , Z. Lu , Nature 2018, 563, 546. 30429610 10.1038/s41586-018-0685-y

[10] H. Conrad , Prog. Mater. Sci. 1981, 26, 123.

[11] X. H. Min , S. Emura , K. Tsuchiya , T. Nishimura , K. Tsuzaki , Mater. Sci. Eng. A 2014, 590, 88.

[12] Y. Chong , R. Gholizadeh , B. Guo , T. Tsuru , G. Zhao , S. Yoshida , M. Mitsuhara , A. Godfrey , N. Tsuji , Acta Mater. 2023, 257, 119165.

[13] Y. Chong , R. Zhang , M. S. Hooshmand , S. Zhao , D. C. Chrzan , M. Asta , J. W. Morris , A. M. Minor , Nat. Commun. 2021, 12, 6158. 34697309 10.1038/s41467-021-26374-w PMC8546145

[14] Y. Chong , M. Poschmann , R. Zhang , S. Zhao , M. S. Hooshmand , E. Rothchild , D. L. Olmsted , J. W. Morris , D. C. Chrzan , M. Asta , A. M. Minor , Sci. Adv. 2020, 6, abc4060. 10.1126/sciadv.abc4060 PMC7608824 33097543

[15] G. Lütjering , J. C. Williams , Titanium, Springer Science & Business Media, Berlin, Germany 2007.

[16] D. Banerjee , J. C. Williams , Acta Mater. 2013, 61, 844.

[17] Y. Zhu , K. Zhang , Z. Meng , K. Zhang , P. Hodgson , N. Birbilis , M. Weyland , H. L. Fraser , S. C. V. Lim , H. Peng , R. Yang , H. Wang , A. Huang , Nat. Mater. 2022, 21, 1258. 36109672 10.1038/s41563-022-01359-2

[18] D. Zhang , D. Qiu , M. A. Gibson , Y. Zheng , H. L. Fraser , D. H. StJohn , M. A. Easton , Nature 2019, 576, 91. 31802014 10.1038/s41586-019-1783-1

[19] Y. Bu , Z. Li , J. Liu , H. Wang , D. Raabe , W. Yang , Phys. Rev. Lett. 2019, 122, 075502. 30848647 10.1103/PhysRevLett.122.075502

[20] S. Wei , K.‐S. Kim , J. Foltz , C. C. Tasan , Adv. Mater. 2024, 36, 2406382. 10.1002/adma.202406382 38842485

[21] C. Wang , D. Yu , Z. Niu , W. Zhou , G. Chen , Z. Li , X. Fu , Acta Mater. 2020, 200, 101.

[22] S. Wei , G. Zhu , C. C. Tasan , Acta Mater. 2020, 206, 116520.

[23] Y. Zheng , R. E. A. Williams , G. B. Viswanathan , W. A. T. Clark , H. L. Fraser , Acta Mater. 2018, 150, 25.

[24] P. Castany , F. Pettinari‐Sturmel , J. Douin , A. Coujou , Mater. Sci. Eng. A 2008, 483–484, 719.

[25] A. Devaraj , V. V. Joshi , A. Srivastava , S. Manandhar , V. Moxson , V. A. Duz , C. Lavender , Nat. Commun. 2016, 7, 11176. 27034109 10.1038/ncomms11176 PMC4821990

[26] M. T. Jia , D. L. Zhang , B. Gabbitas , J. M. Liang , C. Kong , Scr. Mater. 2015, 107, 10.

[27] H. R. Zhang , H. Z. Niu , Y. H. Zhang , M. C. Zang , D. L. Zhang , J. Alloys Compd. 2022, 894, 162517.

[28] H. Wang , Q. Chao , X. Y. Cui , Z. B. Chen , A. J. Breen , M. Cabral , N. Haghdadi , Q. W. Huang , R. M. Niu , H. S. Chen , B. Lim , S. Primig , M. Brandt , W. Xu , S. P. Ringer , X. Z. Liao , Mater. Today 2022, 61, 11.

[29] J. Zhang , Y. Liu , G. Sha , S. Jin , Z. Hou , M. Bayat , N. Yang , Q. Tan , Y. Yin , S. Liu , J. H. Hattel , M. Dargusch , X. Huang , M. X. Zhang , Nat. Commun. 2022, 13, 4660. 35945248 10.1038/s41467-022-32446-2 PMC9363443

[30] J. N. Rousseau , A. Bois‐Brochu , C. Blais , Addit. Manuf. 2018, 23, 197.

[31] L. Liu , C. Chen , R. Zhao , X. Wang , H. Tao , S. Shuai , J. Wang , H. Liao , Z. Ren , Addit. Manuf. 2021, 46, 102142.

[32] Y. Chong , T. Tsuru , B. Guo , R. Gholizadeh , K. Inoue , N. Tsuji , Acta Mater. 2022, 240, 118356.

[33] L. Liu , Q. Ding , Y. Zhong , J. Zou , J. Wu , Y.‐L. Chiu , J. Li , Z. Zhang , Q. Yu , Z. Shen , Mater. Today 2018, 21, 354.

[34] W. Chen , X. Li , S. Jin , L. Yang , Y. Li , X. He , W. Zhang , Y. Wu , Z. Hui , Z. Yang , J. Yang , W. Xiao , G. Sha , J. Wang , Z. Zhou , Nat. Commun. 2023, 14, 8336. 38097587 10.1038/s41467-023-44056-7 PMC10721596

[35] M. Kuzmina , M. Herbig , D. Ponge , S. Sandlöbes , D. Raabe , Science 2015, 349, 1080. 26339026 10.1126/science.aab2633

[36] Y. Chong , R. Gholizadeh , T. Tsuru , R. Zhang , K. Inoue , W. Gao , A. Godfrey , M. Mitsuhara , J. W. Morris Jr. , A. M. Minor , N. Tsuji , Nat. Commun. 2023, 14, 404. 36725856 10.1038/s41467-023-36030-0 PMC9892041

[37] Q. Pan , L. Zhang , R. Feng , Q. Lu , K. An , A. C. Chuang , J. D. Poplawsky , P. K. Liaw , L. Lu , Science 2021, 374, 984. 34554824 10.1126/science.abj8114

[38] H. Mohseni , P. Nandwana , A. Tsoi , R. Banerjee , T. W. Scharf , Acta Mater. 2015, 83, 61.

[39] S. C. Wang , M. Aindow , M. J. Starink , Acta Mater. 2003, 51, 2485.

[40] E. Farabi , P. D. Hodgson , G. S. Rohrer , H. Beladi , Acta Mater. 2018, 154, 147.

[41] Y. W. Chai , H. Y. Kim , H. Hosoda , S. Miyazaki , Acta Mater. 2009, 57, 4054.

[42] K. Prasad , S. Amrithapandian , B. K. Panigrahi , V. Kumar , K. Bhanu Sankara Rao , M. Sundararaman , Mater. Sci. Eng. A 2015, 638, 90.

[43] T. Furuhara , T. Maki , Mater. Sci. Eng. A 2001, 312, 145.

[44] S. L. Semiatin , J. C. Soper , I. M. Sukonnik , Acta Mater. 1996, 44, 1979.

[45] S. Dixit , B. B. Dash , D. Kumar , A. Bhattacharjee , S. Sankaran , Mater. Sci. Eng. A 2023, 873, 144990.

[46] W. Zhu , J. Lei , C. Tan , Q. Sun , W. Chen , L. Xiao , J. Sun , Mater. Des. 2019, 168, 107640.

[47] W. Zhu , Q. Sun , C. Tan , P. Li , L. Xiao , J. Sun , J. Alloys Compd. 2020, 827, 154311.

[48] C. Huang , Y. Zhao , S. Xin , W. Zhou , Q. Li , W. Zeng , J. Alloys Compd. 2017, 693, 582.

[49] D. Qin , H. Liu , Y. Li , Mater. Sci. Eng. A 2023, 867, 144697.

[50] L. Ren , W. Xiao , H. Chang , Y. Zhao , C. Ma , L. Zhou , Mater. Sci. Eng. A 2018, 711, 553.

[51] X. Li , X. N. Wang , K. Liu , G. H. Cao , M. B. Li , Z. S. Zhu , S. J. Wu , J. Mater. Sci. Technol. 2022, 107, 227.

[52] W. Zhu , W. Kou , C. Tan , B. Zhang , W. Chen , Q. Sun , L. Xiao , J. Sun , Mater. Sci. Eng. A 2020, 771, 138611.

[53] Y. Wang , M. Hao , D. Li , P. Li , Q. Liang , D. Wang , Y. Zheng , Q. Sun , Y. Wang , Mater. Sci. Eng. A 2022, 829, 142117.

[54] C. C. Li , C. Xin , Q. Wang , J. Q. Ren , B. Zhao , J. P. Wu , X. L. Pan , X. F. Lu , J. Alloys Compd. 2023, 959, 170497.

[55] V. C. Opini , C. A. F. Salvador , K. N. Campo , E. S. N. Lopes , R. R. Chaves , Mater. Sci. Eng. A 2016, 670, 112.

[56] X. Bao , W. Chen , J. Zhang , Y. Yue , J. Sun , J. Mater. Res. Technol. 2021, 11, 1622.

[57] M. Hao , D. Wang , Y. Wang , T. Zhang , P. Li , Y. Guo , Y. Zheng , Q. Sun , Y. Wang , Acta Mater. 2024, 269, 119810.

[58] H. Zhang , J. Zhang , S. Liu , D. Zhang , G. Liu , J. Sun , Acta Mater. 2023, 255, 119082.

[59] Z. Tarzimoghadam , S. Sandlöbes , K. G. Pradeep , D. Raabe , Acta Mater. 2015, 97, 291.

[60] W. Xu , E. W. Lui , A. Pateras , M. Qian , M. Brandt , Acta Mater. 2017, 125, 390.

[61] C. de Formanoir , G. Martin , F. Prima , S. Y. P. Allain , T. Dessolier , F. Sun , S. Vivès , B. Hary , Y. Bréchet , S. Godet , Acta Mater. 2019, 162, 149.

[62] A. Zafari , M. R. Barati , K. Xia , Mater. Sci. Eng. A 2019, 744, 445.

[63] Y. Chong , T. Bhattacharjee , J. Yi , A. Shibata , N. Tsuji , Scr. Mater. 2017, 138, 66.

[64] Z. Song , X. Zeng , L. Wang , Mater. Res. Lett. 2023, 11, 391.

[65] Z. Yao , M. He , J. Yi , M. Yang , R. Shi , C. Wang , Z. Zhong , T. Yang , S. Wang , X. Liu , Addit. Manuf. 2024, 80, 103969.

[66] Y. Chong , G. Deng , S. Gao , J. Yi , A. Shibata , N. Tsuji , Scr. Mater. 2019, 172, 77.

[67] J. Kim , D. Hall , H. Yan , Y. Shi , S. Joseph , S. Fearn , R. J. Chater , D. Dye , C. C. Tasan , Acta Mater. 2021, 220, 117304.

[68] Y. M. Ren , X. Lin , X. Fu , H. Tan , J. Chen , W. D. Huang , Acta Mater. 2017, 132, 82.

[69] Q.‐q. Zhu , H.‐f. Lan , B.‐s. Lin , D.‐x. Wang , S. Huang , y.‐y. Chen , X.‐d. Yang , J.‐p. Li , J. Alloys Compd. 2023, 968, 171964.

[70] K. Wijesinghe , C. Herath , J. G. Michopoulos , S. M. Arnold , A. Achuthan , Acta Mater. 2024, 276, 120080.

[71] T. Chen , H. Z. Lu , W. S. Cai , L. H. Liu , Z. Wang , C. Yang , Scr. Mater. 2023, 236, 115676.

[72] J. A. Lee , J. Park , M. J. Sagong , S. Y. Ahn , J.‐W. Cho , S. Lee , H. S. Kim , Nat. Commun. 2025, 16, 931. 39843413 10.1038/s41467-025-56267-1 PMC11754831

[73] F. Cao , K. S. R. Chandran , P. Kumar , Scr. Mater. 2017, 130, 22.

[74] M. Jia , D. Zhang , J. Liang , B. Gabbitas , Metall. Mater. Trans. A 2017, 48, 2015.

[75] H. R. Zhang , H. Z. Niu , M. C. Zang , J. K. Yue , D. L. Zhang , Mater. Sci. Eng. A 2020, 770, 138570.

[76] Y. Dong , D. Wang , Q. Li , X. Luo , J. Zhang , K. G. Prashanth , P. Wang , J. Eckert , L. Mädler , I. V. Okulov , M. Yan , Mater. Today Adv. 2023, 17, 100347.

[77] A. Issariyapat , P. Visuttipitukul , T. Song , J. Umeda , M. Qian , K. Kondoh , Mater. Sci. Eng. A 2020, 779, 139136.

[78] C. H. Ng , M. J. Bermingham , M. S. Dargusch , Addit. Manuf. 2021, 39, 101855.

[79] S. L. Lu , J. H. Wang , Y. Y. Sun , T. Song , M. Qian , Scr. Mater. 2022, 212, 114578.

[80] S. Wang , Z. Hu , Z. Huang , B. Gao , X. Chen , J. Hu , Y. Zhu , Y. Li , H. Zhou , Int. J. Plast. 2024, 174, 103908.

[81] M. L. Wasz , F. R. Brotzen , R. B. McLellan , A. J. Griffin , Int. Mater. Rev. 1996, 41, 1.

[82] J.‐W. Zhang , I. J. Beyerlein , W.‐Z. Han , Phys. Rev. Lett. 2019, 122, 255501. 31347895 10.1103/PhysRevLett.122.255501

[83] Y.‐M. Kim , B.‐J. Lee , Acta Mater. 2008, 56, 3481.

[84] S. Plimpton , J. Comput. Phys. 1995, 117, 1.

[85] A. Stukowski , B. Sadigh , P. Erhart , A. Caro , Modell. Simul. Mater. Sci. Eng. 2009, 17, 075005.

