# Supplementary material for: Harnessing Bifunctional Nitrogen‐Dislocation Interactions for a Record Ultra‐Strong‐and‐Ductile Duplex Titanium Alloy
Source: Adv Sci (Weinh). 2025 Jun 19;12(30):e02349. doi: 10.1002/advs.202502349 (PMC12376552; doi:10.1002/advs.202502349)
Supplement: Supplementary file 1 — Supporting Information [file ADVS-12-e02349-s001.docx]

**Supplementary Information for**

**Harnessing Bifunctional Nitrogen‐Dislocation Interactions for a Record Ultra‐Strong‐and‐Ductile Duplex Titanium Alloy**

*Chongle Zhang,* *Xuanzhe Li, Suzhi Li, Jinyu Zhang*, Gang Liu, Jun Sun**

State Key Laboratory for Mechanical Behavior of Materials, Xi'an Jiaotong University, Xi'an 710049, People's Republic of China

E-mail address: [jinyuzhang1002@xjtu.edu.cn](mailto:jinyuzhang1002@xjtu.edu.cn) (Jinyu Zhang);

junsun@mail.xjtu.edu.cn (Jun Sun)

**The file includes:**

Supplementary Texts 1-3

Supplementary Figs. 1-20

Supplementary Tables. 1-6

Additional references

**Supplementary Text 1.** Alloy composition design

In this study, The composition of Ti-2.8Cr-4.5Zr-5.2Al-0.4N was designed to synergize martensitic phase transformation, solution strengthening, and interstitial hardening, guided by the following principles: (i) The slowly eutectoid yet fast diffusive β-stabilizer Cr can refine martensites and increase hardenability during WQ.^[1]^ Add Cr element about ~2.8 wt.% so that molybdenum equivalent ([Mo]eq) is about ~3.5,^[2]^ below the α′/α′ critical threshold ([Mo]eq ~4),^[3]^ which allows the alloy to obtain the highest solution strengthening while ensuring that forms harder α′ martensite rather than the soft α′′ martensite during quenching.^[3]^ (ii) Al as the α-stabilizer can also strengthen the alloy, especially the HCP-α′ martensites. To avoid the excessive Al element forming Ti_3_Al brittle phase to deteriorate the ductility of the alloy, the content of Al element is controlled at 5.2 wt.% in this work. (iii) The neutral element Zr is an infinitely solid solution in both α-Ti and β-Ti alloys, which can improve strength without reducing plasticity. Given the addition of Zr would weaken the stabilizing effect of α-stabilizers but enhance the effectiveness of β-stabilizers,^[4]^ the Zr content was optimized to 4.5 wt.% to avoid its influence on the martensitic transformation.^[5]^ (iv) For the commonly incorporated interstitial solutes N, O, and C in Ti alloys,^[6,7]^ N was prioritized due to its superior interstitial hardening capacity and precise compositional controllability, enabling effective tailoring of N-dislocation interactions during high-temperature processing to engineer the final microstructures. Systematic N-content optimization within the Ti-Cr-Zr-Al alloy system revealed that 0.4 wt.% N optimally balances microstructure and mechanical performance by controlled interstitial atom-dislocation interactions (Fig. S6, Supporting Information).

**Supplementary Text 2.** Thermomechanical processing

In this study, the β-transus temperatures (T*_β_*) of base, 0.3N, 0.4N and 0.5N alloys determined by the metallographic method 950 ± 5℃, 1000 ± 5℃, 1040 ± 5℃ and 1080 ± 5℃, respectively. Homogenization was performed at 1200 ℃ for 60 min in an Ar atmosphere followed by water quenching, and then cut into rectangular slabs with a thickness of 10 mm. To obtain similar microstructures (i.e., heterogeneous lamella structure Ti alloys), the homogenized specimens were processed and heat-treated in the same way based on the T*_β_*, that is, the cyclic hot-rolling & short-time-solution (HR&SS) processing, see Route-I in Fig. S1 (Supporting Information). First, the samples were heated to ~20° below the T*_β_* and held for 5 min, and then the samples were rolled at ~20° below the T*_β_*. After each rolling pass with a reduction in thickness of 10%, the specimen was heated to the furnace temperature (below the T*_β_* ~20 ℃) for ~1 min. Here, the volume fraction and morphology of the primary α_p_ grains in the final alloy microstructure were precisely regulated by controlling the hot rolling and solutioning temperature below the T*_β_* ~20 ℃. This is because α_p_ tends to spheroidize when the rolling and solutioning temperature is too high, and it is difficult to form the elongated α_p_ morphology.^[8]^ When the temperature is too low, too many α_p_ grains will precipitate to increase the phase stability of metastable β matrix, making it difficult to undergo martensitic transformation.^[9]^ On the other hand, the α_p_ grains with appropriate volume fraction in the final microstructure can be adapted to the plastic deformation during the tensile test, which helps to improve the mechanical properties of the alloy.^[10]^ The HR&SS processes were repeated until the total thickness reduction of the specimen reached 80%. The cycles 8 times and 80% total reduction are mainly to introduce high-density dislocations in the α_p_ phase and β matrix, which provide the structural basis for the subsequent formation of LAGBs and α′-NTNMs in α_p_ phase and β matrix, respectively. Finally, the HR&SS sample was water quenching (WQ) and air cooling (AC) to form the desired heterogeneous lamella structure Ti alloy, hereafter denoted as LML-WQ and LML-AC alloys, respectively.

To confirm the critical roles of LAGBs and coherent PBs, we rapidly heated the heterogeneous lamella structure Ti alloys obtained from Route-I to rolling/solutioning temperatures (below T_β_ ~20 ° C) for 1 minute to diminish N-dislocation interactions, followed by AC or WQ to RT, see Route-II in Fig. S1 (Supporting Information), hereafter denoted as LM-AC and LM-WQ 0.4N alloys, respectively.

**Supplementary Text 3.** Theoretical calculations of strengthening contributions

The present LML-WQ 0.4N alloy shows heterogeneous microstructure, *i.e.*, soft α_p_ and hard β*_trans_*. Based on the hetero-deformation-induced (HDI) strengthening caused by geometrically necessary dislocations (GNDs), we apply a modified model to estimate the yield strength of LML-WQ 0.4N alloys,^[11,12]^ *i.e.*,

${}_{y}$ =$f_{\alpha}{}_{\alpha}$ + $f_{\beta t}{}_{\beta t}$ + ${}_{HDI}$ (1)

${}_{\alpha}$ = ${}_{0\alpha}$ + ${}_{P\alpha}$ (2)

${}_{\beta t}$ = $={}_{0\beta t}$ + ${}_{P\beta t}$ (3)

where *σ_α_* and *σ_βt_* are the stresses at the yielding point of α and β*_trans_* microstructure, respectively, and $f_{\alpha}$ and $f_{\beta t}$ are their corresponding volume fractions. ${}_{0}$ is the lattice friction stress, including the critical resolved shear stress (CRSS) of pure Ti (σ_CRSS_ ∼180 MPa),^[13]^ substitutional solid solution strengthening (σ_SS_)^[13,14]^ and interstitial solid solution strengthening (σ_IS_),^[7,15]^ while the subscript *α* and *βt* indicate the α and β*_trans_* microstructure, respectively.

Here, the lattice friction stress $\sigma_{0}$ of the LML-WQ 0.4N Ti alloy, including the solid solution strengthening can be calculated by the following equation:^[1,13]^

${}_{0}= {}_{CRSS}$ + ${}_{SS}$ + ${}_{IS}$ (4)

$\sigma_{\mathrm{SS}}$= $f_{\alpha}$ ${(\sum_{i} {B_{i}}^{3/2}X_{i\alpha})}^{2/3}$ + $f_{\alpha} {(\sum_{i} {B_{i}}^{3/2}X_{i\alpha})}^{2/3}$ + $f_{\beta} {(\sum_{i} {B_{i}}^{3/2}X_{i\beta})}^{2/3}$ (5)

${}_{IS}=f_{\alpha}(0.02\mu({2c_{N\alpha})}^{1/2})$ + $f_{\alpha}(0.02\mu({2c_{N\alpha})}^{1/2})$ + $f_{\beta}(0.02\mu(2c_{N\beta})^{1/2})$ (6)

where *X_iα_*, *X_iα′_* and *X_iβ_* are the atomic concentrations of the solute *i* in α, α′ and β phases, respectively. *B_i_* is the strengthening coefficient for the solute *i.*^[1,13]^ *μ* is the shear modulus (Table S5, Supporting Information). *c_Nα_, c_Nα′_, c_Nβ_* are the N interstitial solutes in α, α′ and β phases. Similarly, the lattice friction stress ${}_{0\beta t}$ of the β*_trans_* microstructure can be expressed as:

${}_{0\beta t}$ =$f_{\alpha}{}_{0\alpha}$ + $f_{\beta}{}_{0\beta}$ (7)

where $f_{\alpha}$ and $f_{\beta}$ are the volume fractions of α′ and β phases (Table S5, Supporting Information**)**, while the subscript *α′* and *β* indicate the α′ and β phases, respectively. Based on the APT results in Table S4 (Supporting Information), the strength contribution of ${}_{0}$ is ~620 MPa.

In addition, $\sigma_{P}$ is the hetero-phase interface strengthening. Since there is no precipitate inside α_p_ grains, the ${}_{P\alpha}$ is simply taken as 0 MPa. By contrast, for the β*_trans_* microstructure, the interface strengthening is mainly from the α′/α′ TBs and α′/β PBs. Note that the α′/α′ and α′/β interface strengthening often follows the Hall-Petch (H-P) relationship.^[16,17]^ Because dislocations initially propagate inside the soft phase and then pile up at the PB, the strength is determined by the thickness and properties of the soft phase. In the β*_trans_* structure of the LML-WQ 0.4N alloy, compared with the N-enriched α′ lamellae, BCC β lamellae are the soft phase.^[18]^ Thus, the strength contributed by PBs can be evaluated by:^[19,20]^

$\sigma_{P\beta t}$ = $k_{L}{(L_{\beta})}^{-1/2}$ (8)

where $k_{L}$ is the H-P coefficient and *L_β_* is the average thickness of the β lamellae (~32 ± 8 nm). The H-P coefficient $k_{L}$ of the phase boundary can be calculated by:

$k_{L}$ = ${(\frac{n^{2}\mu^{2}b^{2}}{8L_{\beta}})}^{1/2}$ (9)

where *n* = ~3 is the number of dislocations crossing the same lamellae (obtained through TEM analysis of the small strained sample, see Fig. 5g in main text), and *b* is the Burgers vector (Table S5, Supporting Information). Here, the PB strengthening is estimated to be 272 MPa.

Finally, ${}_{HDI}$ is the HDI strengthening. For homogeneous materials, dislocation strengthening, also known as Taylor hardening, is the primary hardening mechanism while straining.^[12]^ However, in heterogeneous materials, it has been reported that HDI hardening is rather prominent and even much higher than Taylor hardening.^[12,21,22]^ Furthermore, upon yielding point, soft zones deform plastically while hard zones remain elastic, in consequence, GNDs generated in the former to sustain plastic strain. However, soft zones are surrounded by hard zones, impeding free movements of GNDs across the zone boundaries/interfaces (such as grain boundaries) in this tensile strain. Therefore, some recent studies have included dislocation strengthening and grain boundary strengthening as part of HDI strengthening.^[11,21-23]^ In practice, the contribution of the HDI stress to the total flow stress is usually estimated from the loading-unloading-reloading (LUR) tensile curve, see Fig. S14a (Supporting Information). The measured ${}_{\mathrm{HDI}}$ is ∼662 MPa when the sample is yielding, see Fig. S14b (Supporting Information). Using these data in Table S5 (Supporting Information), the theoretical calculation (Cal.) of σ_y_ = 1524 MPa agrees very well with the measured (Exp.) σ_y_ = 1532 MPa for the LML-WQ 0.4N alloy, see Fig. S15 (Supporting Information).


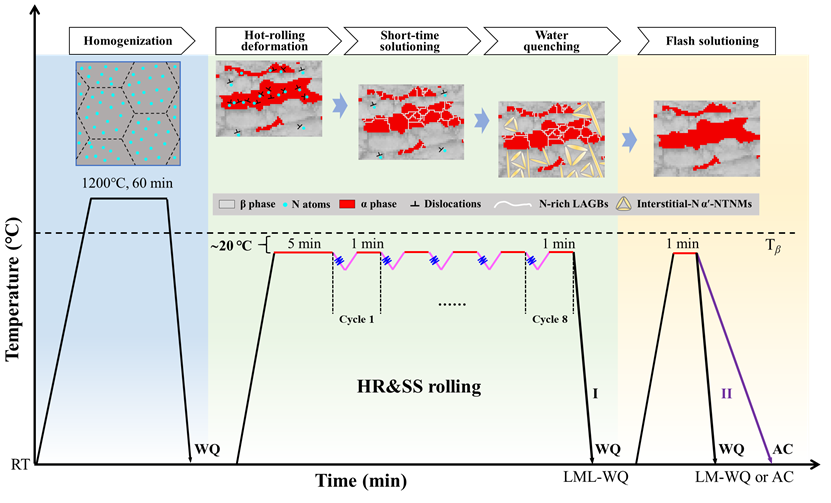


**Fig. S1 |** The thermomechanical processing scheme of LML-WQ, LM-WQ and LM-AC Ti alloys. By combining the total rolling reduction and duration of the solution treatment, the lamellar duplex Ti alloys consisting of elongated α_p_ grains decorated with N-rich low-angle grain boundaries (LAGBs) and β*_trans_* structure with coherent interstitial-N α′-nanotwinned martensites (α′-NTNMs) were prepared. Route-I: the processing route to prepare LML-WQ 0.4N Ti alloys, and Route-II: the processing route to prepare LM-AC and LM-WQ 0.4N Ti alloys. Moreover, to clearly characterize the types of pre-dislocations produced by the HR&SS process, we reduce the cooling rate (~100 ℃/s) in the last step of the HR&SS process to prevent the martensitic transformation, and then WQ when the temperature drops to 500-600 ℃ to inhibit more α precipitation. For LML-WQ 0.4N alloys, although the α phase is precipitated from the β matrix, a part of the β-matrix remains at room temperature without diffusion (β-to-α) transformation and diffusionless displacive (β-to-α′) transformation, which facilitates the determination of dislocations caused by high temperature deformation.


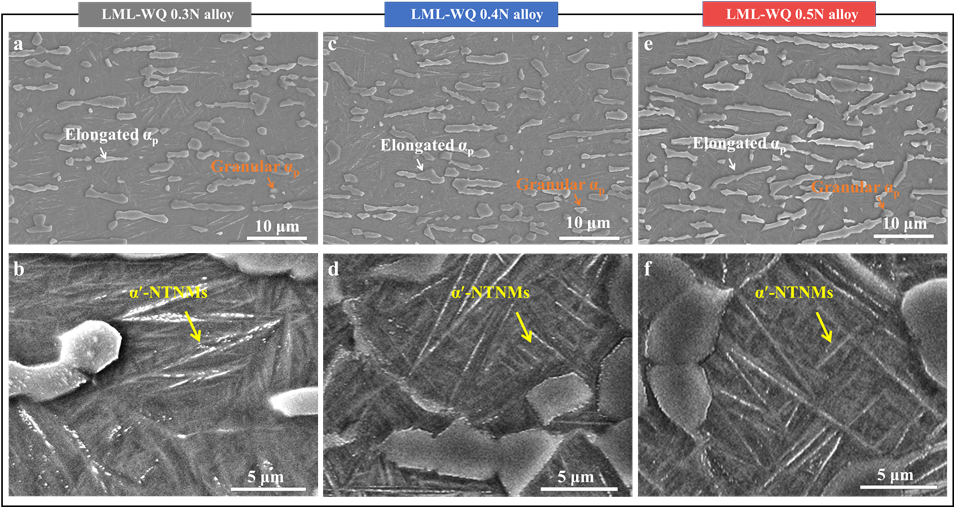


**Fig. S2 |** The heterogeneous lamella structures of the LML-WQ 0.3N, LML-WQ 0.4N and LML-WQ 0.5N alloys. a-b) SEM images of LML-WQ 0.3N alloy. c-d) SEM images of LML-WQ 0.4N alloy. e-f) SEM images of LML-WQ 0.5N alloy.


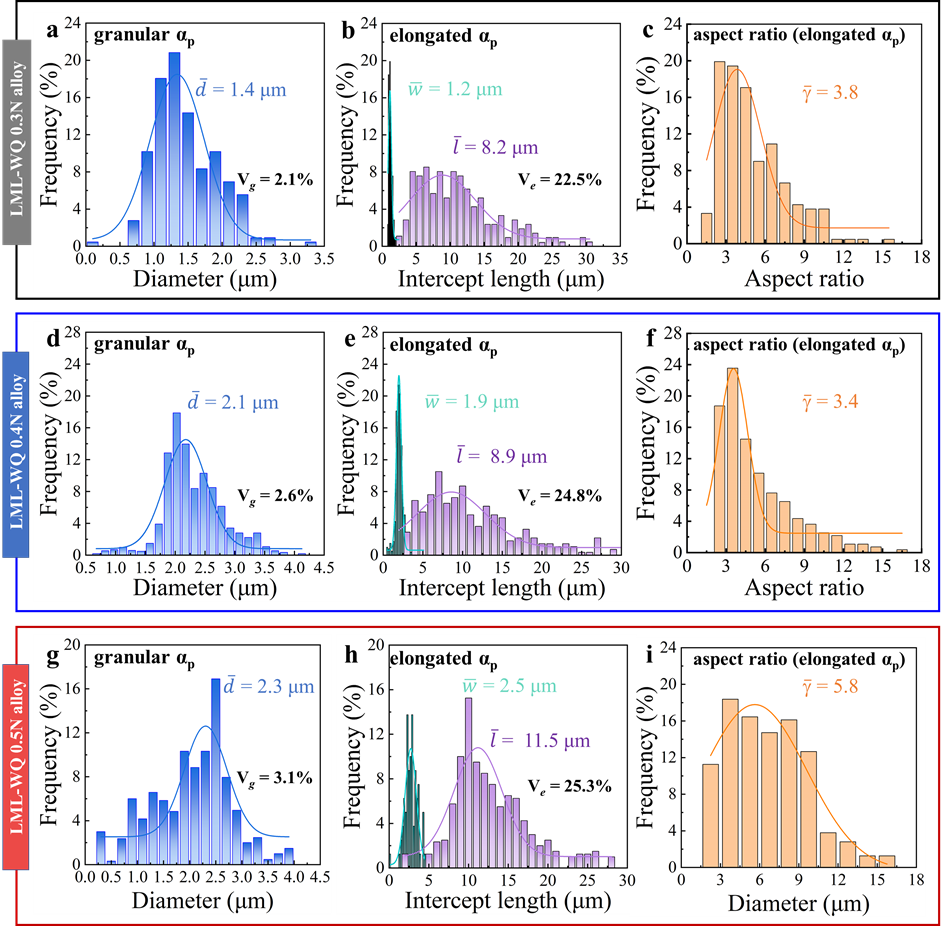


**Fig. S3 |** The statistical information of α_p_ grains (including granular α_p_ and elongated α_p_ grains) in the LML-WQ 0.3N, LML-WQ 0.4N and LML-WQ 0.5N alloys. a-c) LML-WQ 0.3N alloy. d-f) LML-WQ 0.4N alloy. g-i) LML-WQ 0.5N alloy.


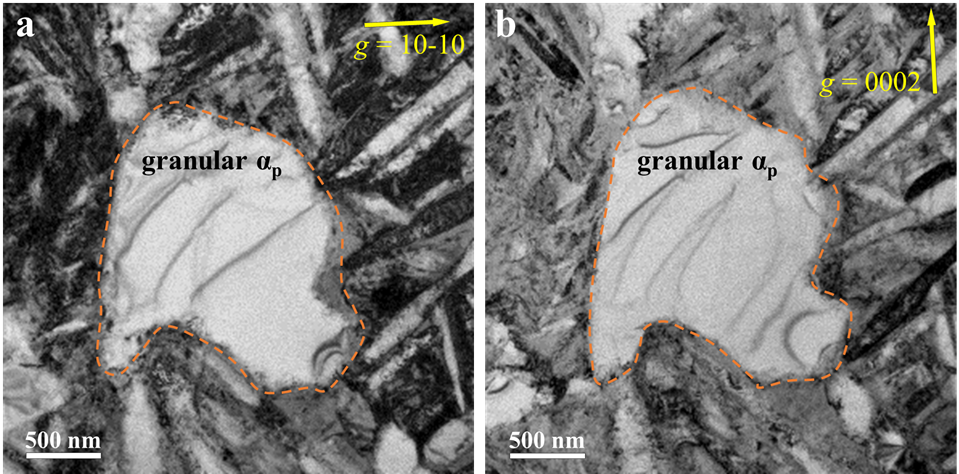


**Fig. S4 |** TEM characterization of granular α_p_ in the LML-WQ 0.4N alloy. a-b) Two-beam condition analysis showing the granular α_p_ grain without dislocations, indicating that the granular α_p_ grain has completely recrystallized.


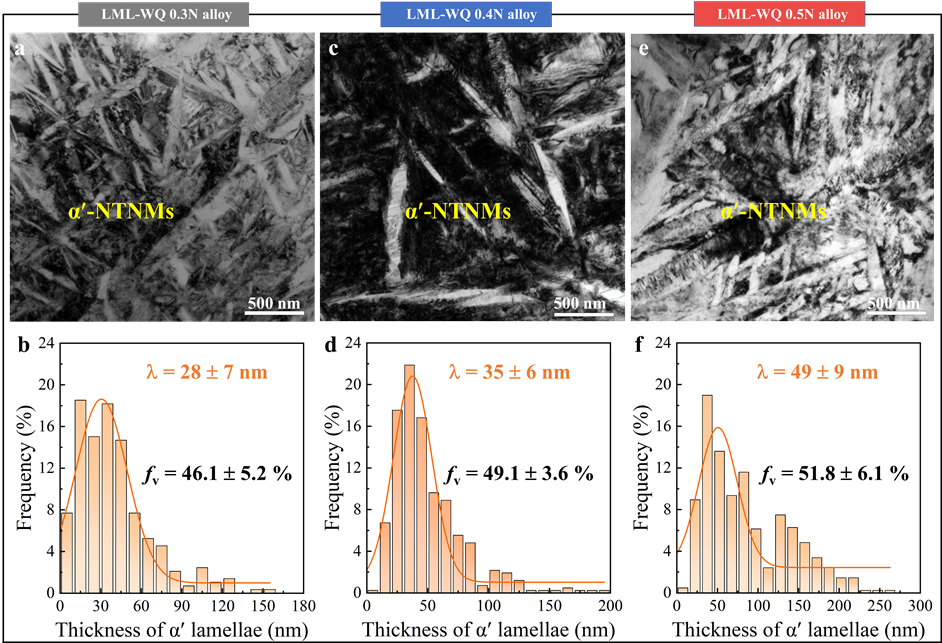


**Fig. S5 |** The β_trans_ microstructure of the LML-WQ 0.3N, LML-WQ 0.4N and LML-WQ 0.5N alloys. a-b) The BF-TEM image and thickness of α′ lamellae in LML-WQ 0.3N alloy. c-d) The BF-TEM image and thickness of α′ lamellae in LML-WQ 0.4N alloy. e-f) The BF-TEM image and thickness of α′ lamellae in LML-WQ 0.5N alloy.


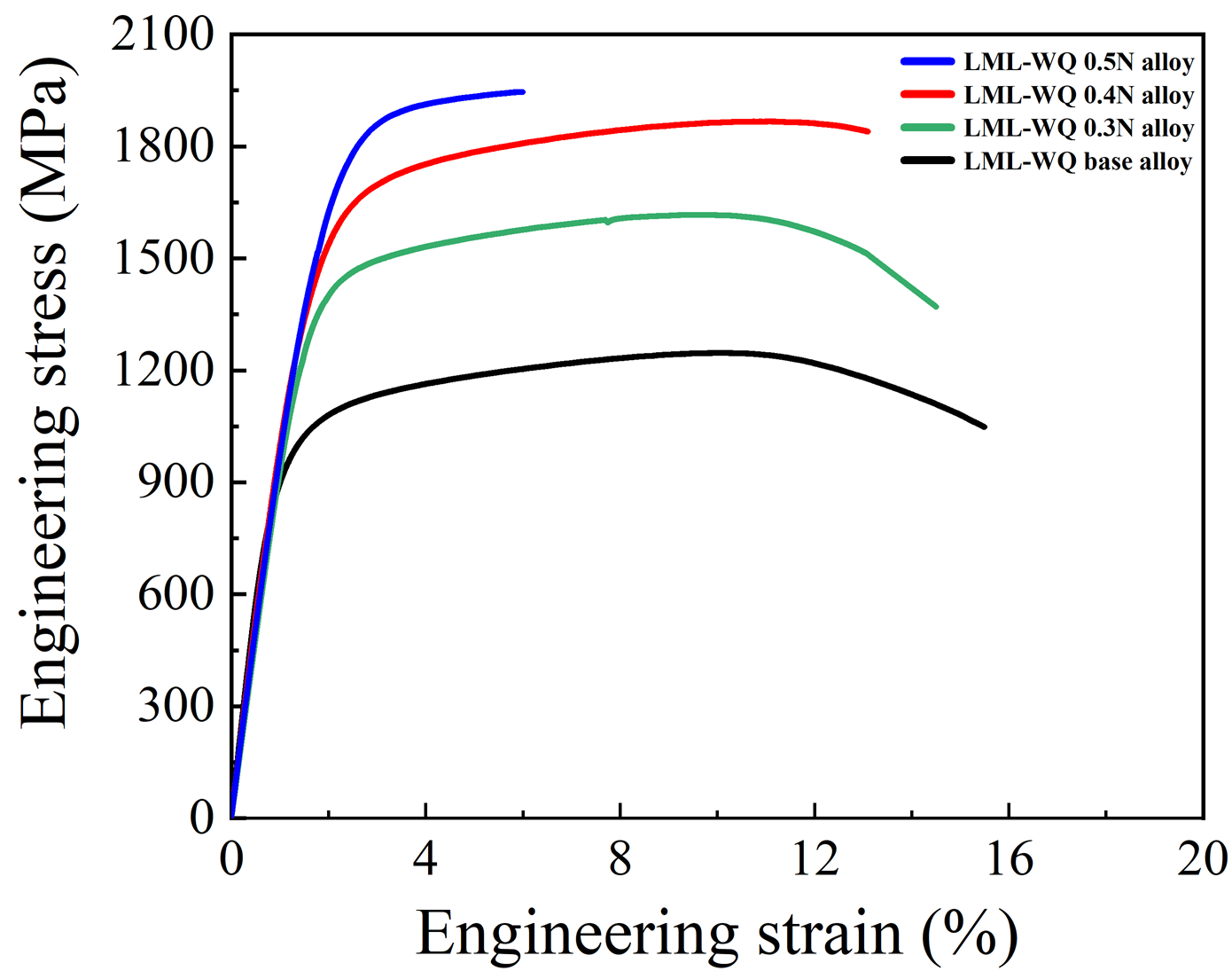


**Fig. S6 |** Engineering stress-strain curves for the LML-WQ Ti alloys with different N content.


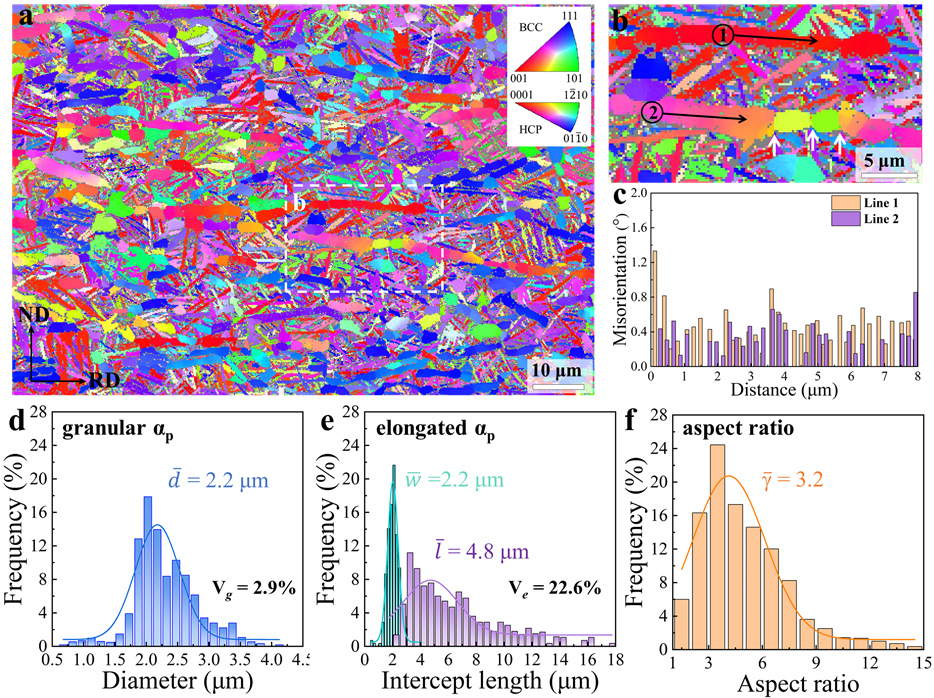


**Fig. S7 |** Microstructures of the LM-AC 0.4N alloy. a) The EBSD inverse pole figure (IPF) showing the lamellar structure composed of two different morphologies of α grains, and β*_trans_* structure. b) The EBSD image taken from the area marked in (a), demonstrating the elongated and granular α_p_ grains. c) The corresponding point-to-point misorientation angle variation, measured with respect to the origin, across elongated α_p_ grains along the black arrows in (b). High angle grain boundaries (HAGBs) as marked by white arrows in (b). d) Histograms showing the sizes of granular α_p_ grains. e-f) Histograms showing the sizes and aspect ratios of elongated α_p_ grains.


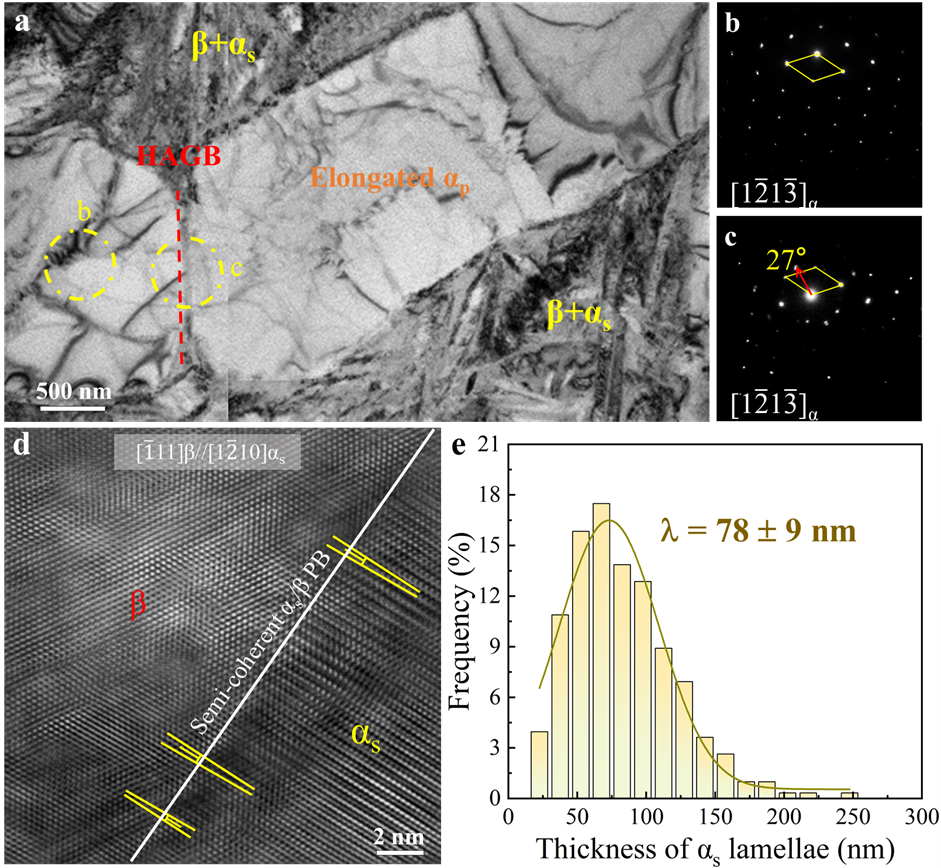


**Fig. S8 |** Microstructure of the LM-AC 0.4N alloy. a) A Bright-field TEM image showing elongated α_p_ grain and β*_trans_* structure, where the elongated α_p_ grain without LAGBs, and HAGB was marked by the red dotted line. b-c) The corresponding selected-area electron diffraction from the area marked in (a). d) The HR-TEM image showing the semi-coherent α_s_/β PB with misfit dislocations, these dislocations are marked by yellow lines. e) Statistical distribution for the thickness of α_s_ lamellae in the β*_trans_* structure


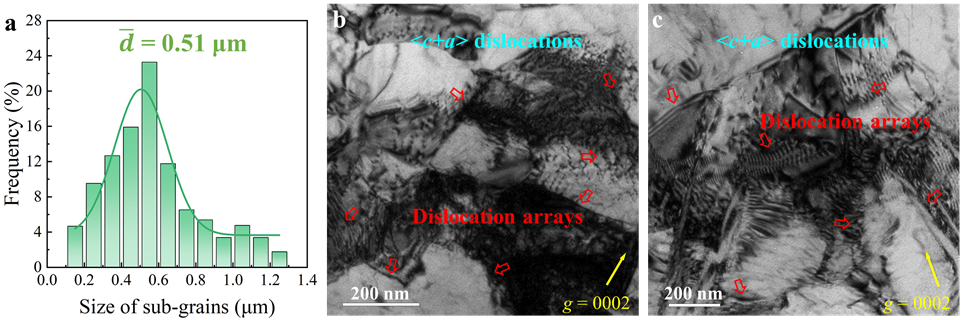


**Fig. S9 |** The microstructure of elongated α_p_ grains in the LML-WQ 0.4N alloy. a) Histograms showing the sizes of sub-grains. b-c) Bright-field TEM images showing that LAGBs in elongated α_p_ grains are composed of dislocation arrays. Due to <***a***> dislocations being invisible under ***g*** = (0002), these LAGBs are mainly characterized by pyramidal <***c***+***a***> dislocations.


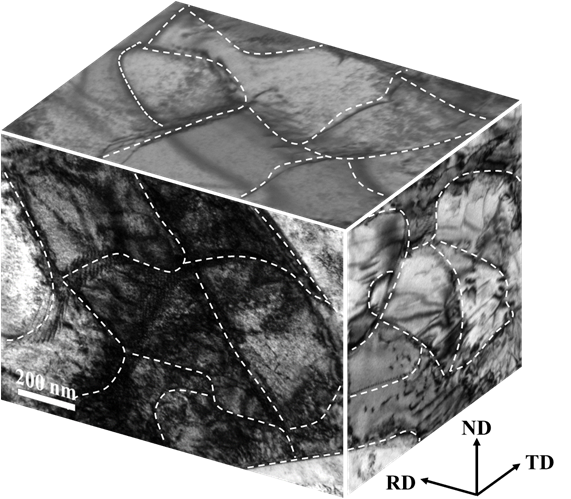


**Fig. S10 |** Microstructures of the LML-WQ 0.4N alloy. A representative three-dimensional (3D) BF-TEM image showing the formation of 3D LAGB networks in elongated α_p_ grains. ND is the normal direction, TD is the transverse direction, and RD is the rolling direction.

**
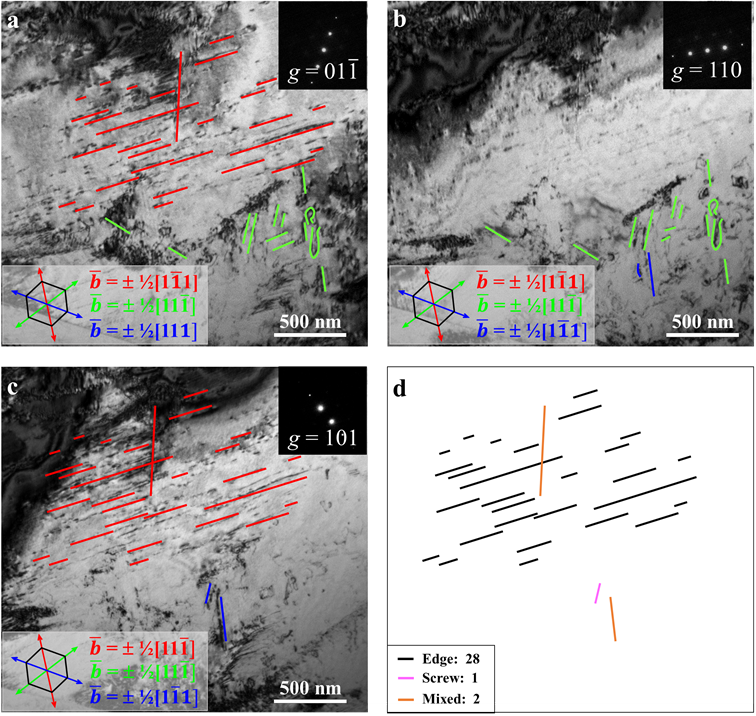
**

**Fig. S11 |** TEM two-beam analysis of dislocations in a local area of the LML-WQ 0.4N alloy under a moderate cooling rate. a) The beam direction is close to <$\bar{\text{1}}$11>_β_ and diffraction vector is ***g*** = (01$\bar{\text{1}}$). b) The beam direction is close to <$\bar{\text{1}}$11>_β_ and diffraction vector is ***g*** = (110). c) The beam direction is close to <011>_β_ and diffraction vector is ***g*** = (101). d) Schematic drawings of dislocation types. (black: edge dislocations, pink: screw dislocations, and orange: mixed dislocations). The number of edge dislocations occupied more of all measured dislocation lines (28 of 31, or ~90%), suggesting that the pre-generated dislocations by HR&SS processes were dominated by edge dislocations. A schematic showing the Burgers vectors of dislocations, as highlighted by different colors.


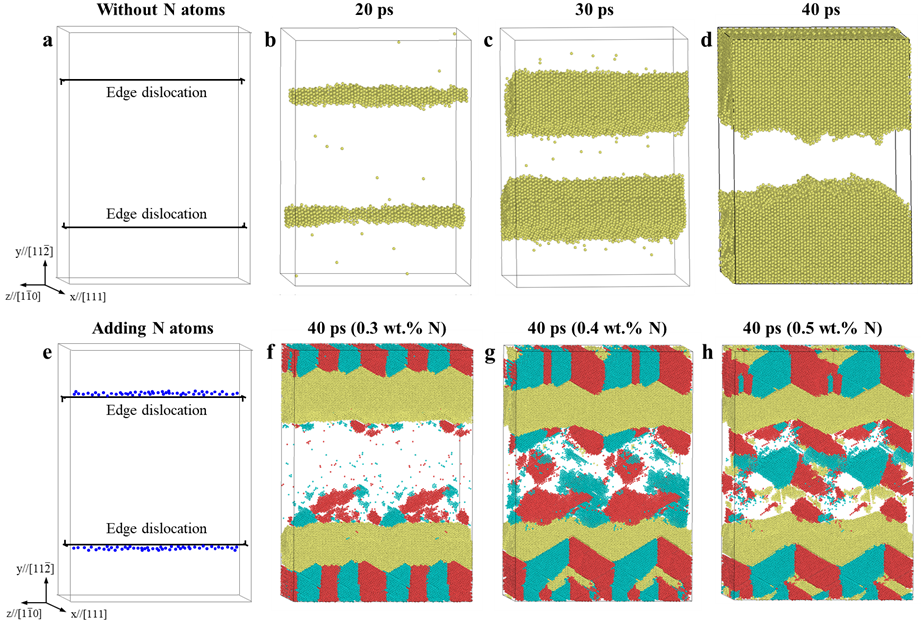


**Fig. S12 |** Atomistic simulations of the formation of nanotwinned nanomartensite with the aid of nitrogen-edge dislocation interactions. a) ½<111>_β_ edge dislocation dipole in a pure β simulation cell. b) One α′-martensite is observed to preferably nucleate from the edge dislocation line when three-dimensional tensile stress is applied to the supercell. c-d) One α′-martensite gradually grows with a loading time of 30 ps and 40 ps. e) ½<111>_β_ edge dislocation dipole with N-segregation in a pure β simulation cell. f-h) Three α′-martensite variants are observed to preferably nucleate from the edge dislocation with different N-segregation content when three-dimensional tensile stress is applied to the supercell, a loading time of about 40 ps. The three α-variants are shown by yellow, red and cyan colors, respectively.

**
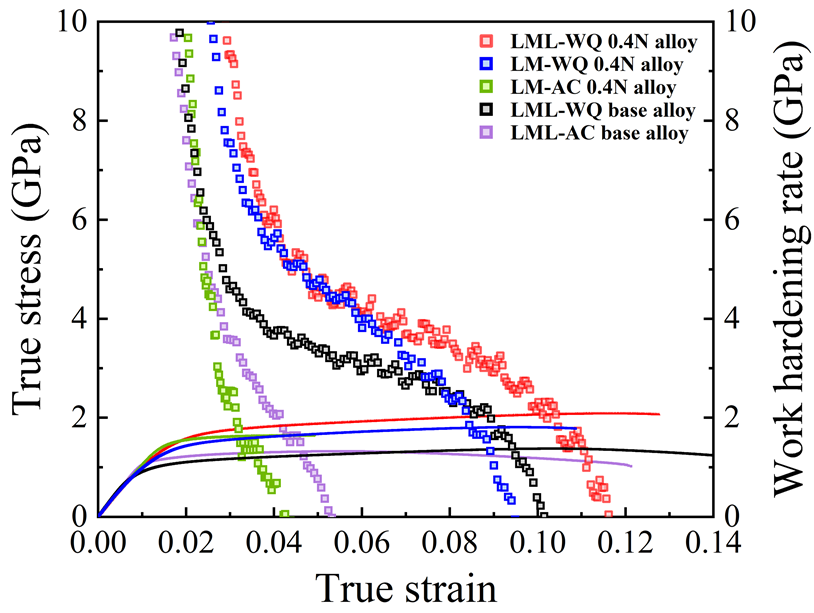
**

**Fig. S13 |** The work-hardening rate vs true strain curves of the base and N-doped alloys. The LM-AC 0.4N alloys exhibit one-stage strain hardening behavior, showing the work hardening rate (WHR, Θ) monotonically drops with increasing plastic strains. It appears that the LML-WQ 0.4N alloy has the maximum WHR Θ in the entire plastic deformations stage. The WHR Θ is higher than 2 GPa before necking, thus showing significant work hardening capability for large uniform elongation..


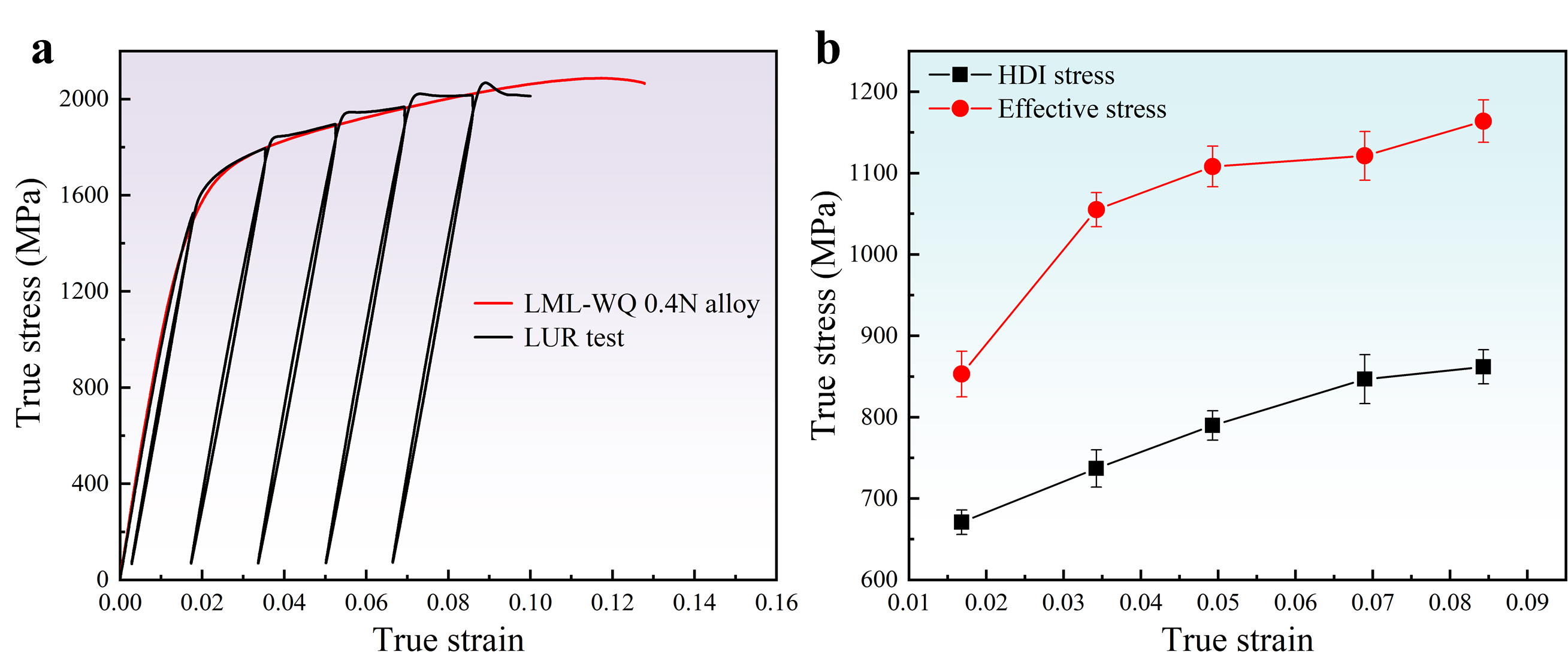


**Fig. S14 |** The stress partitioning behavior of the LML-WQ 0.4N alloy. a) Loading-unloading-reloading (LUR) tests for the LML-WQ 0.4N alloy. b) The evolutions of HDI and effective stresses with the true strain. Error bars in (b) indicate the standard deviations for three tests.


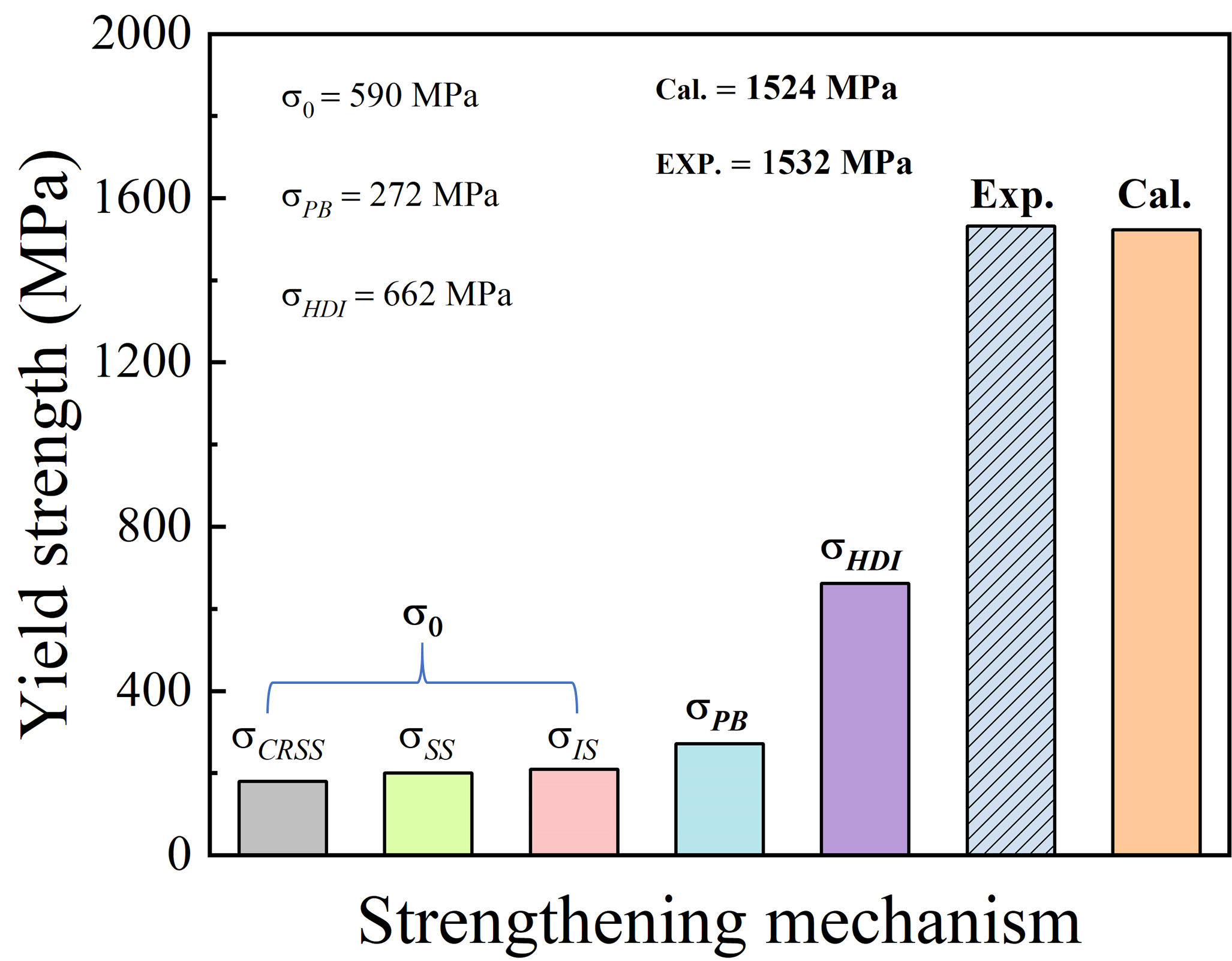


**Fig. S****15 |** Contributions to the yield strength of each strengthening mechanism of the LML-WQ 0.4N alloy. Taking the parameters listed in Table S5, the theoretical calculation (Cal.) of σ_y_ = 1524 MPa agrees well with the measured (Exp.) σ_y_ = 1532 MPa for the LML-WQ 0.4N alloy.


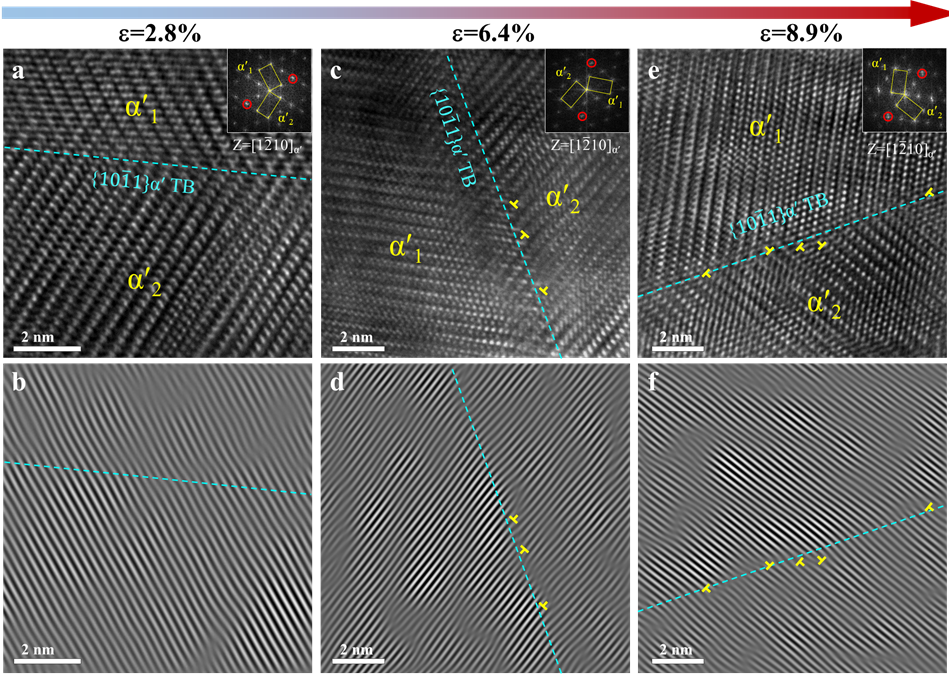


**Fig. S16 |** Deformation microstructure of the α′/α′ twined boundary in the LML-WQ 0.4N alloy. a, c, e) HR-TEM images showing the atomic-level α′/α′ twinned boundary at tensile strains of about 2.8%, 6.4% and 8.9%, respectively. b, d, f) The corresponding images of lattice fringes obtained by mask red circles for (a), (c) and (e) respectively, showing more dislocations storage in the coherent twin boundary (TB) with increasing plastic strains. The interface dislocations, as marked by the yellow symbol of “**⊥**”.


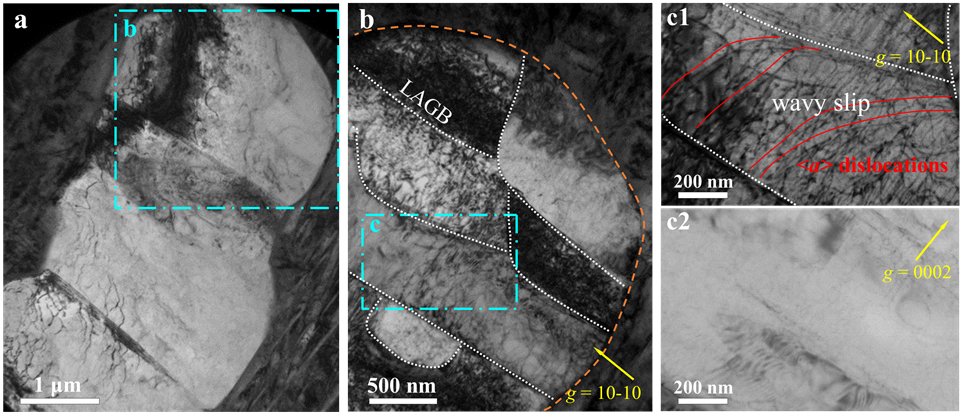


**Fig. S17 |** The BF-TEM images of the deformation substructures of the elongated α_p_ grain at tensile strains of ~6.4% in LML-WQ 0.4N alloys. a-b) BF-TEM images demonstrating some sub-grain in elongated α_p_ grain after tensile strains of ~6.4%, and the LAGBs are marked by white dotted lines. c1-c2) Two-beam condition analyses show that a large number of <***a***> inside with wavy morphology inside the α_p_ grains are activated.

**
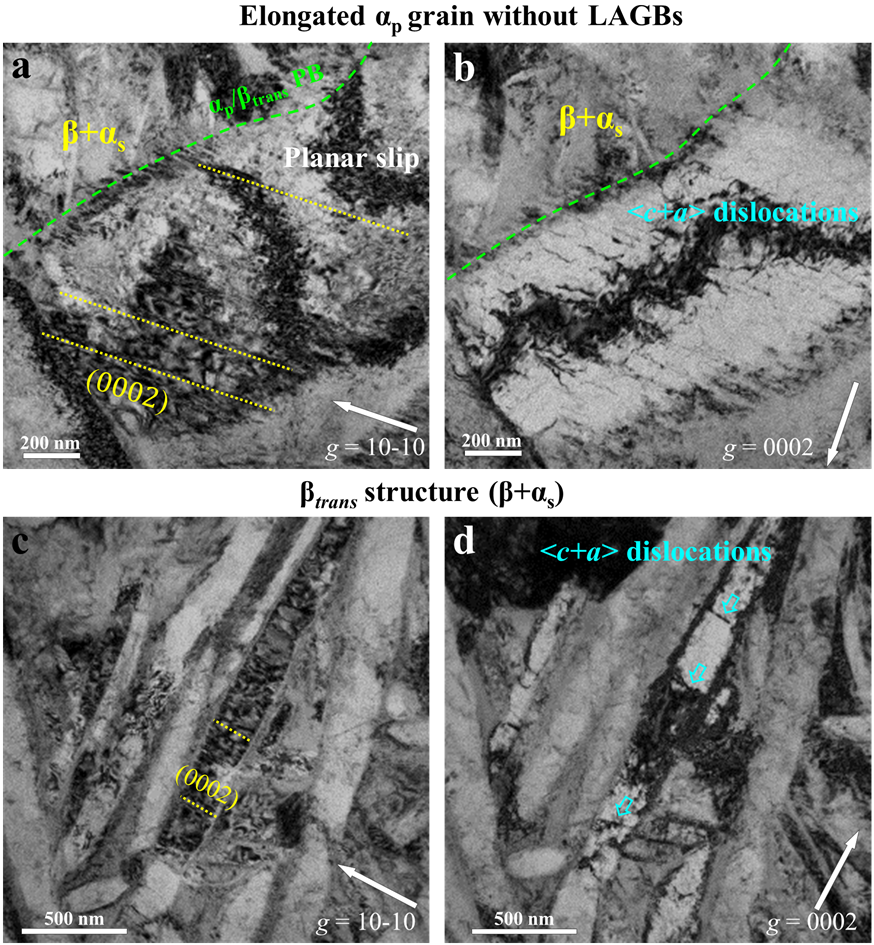
**

**Fig. S18 |** TEM characterization of dislocations in LM-AC 0.4N alloys deformed by fracture strain. a, b and c, d) Two-beam condition analyses of dislocations in elongated α_p_ grain and α_s_ lamella, respectively. Well-aligned planar dislocation-slip along (0002) basal planes was activated. The density of <***c+a***> dislocations was significantly lower than in the LML-WQ 0.4N alloy.


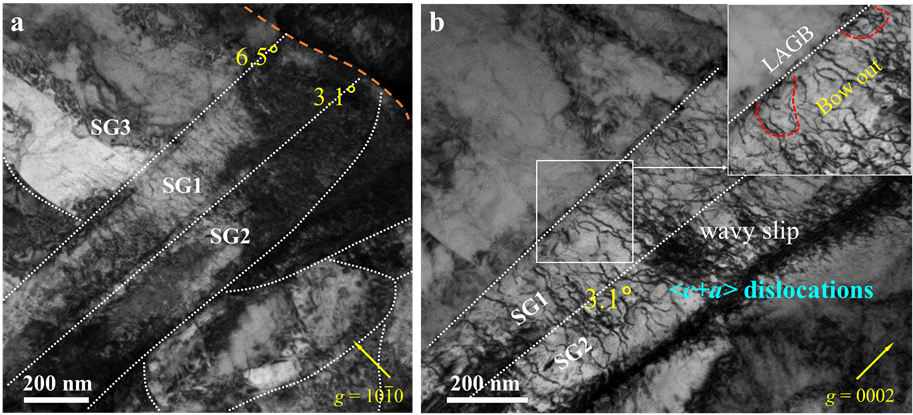


**Fig. S19 |** The BF-TEM images of the deformation substructures of the elongated α_p_ grain at tensile strains of ~8.9% in LML-WQ 0.4N alloys. a) TEM images demonstrating some sub-grain in elongated α_p_ grain, and the LAGBs are marked by white dotted lines. b) TEM image shows a large number of <***c****+****a***> dislocations with bow-out shapes near an LAGB inside the elongated α_p_ grains.

**
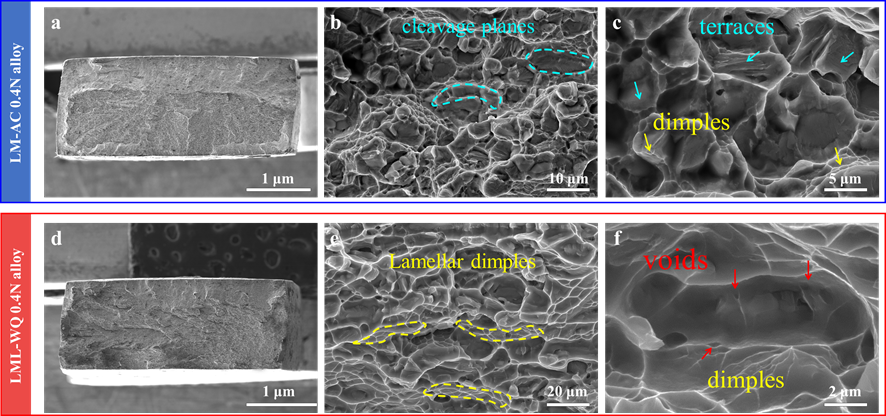
**

**Fig. S20 |** Fracture morphologies of the LM-AC 0.4N and LML-WQ 0.4N alloys. a) Projection of the entire fracture surface of the LM-AC 0.4N alloy. SEM images of fracture surfaces at (b) low and (c) high magnifications, showing terraces and dimples, *i.e.*, mixture of ductile and brittle features. d) Projection of the entire fracture surface of the LML-WQ 0.4N alloy. SEM images of fracture surfaces at (e) low and (f) high magnifications, showing voids and dimples, *i.e.*, a ductile feature.

**Table S1.** Five possible axis/angle misorientations between the 12 variants of the Burgers orientation relationship that could form from each parent β grains.^[24-27]^

| **Unique variant** | **Rotation angle** | **Rotation axis** |
| --- | --- | --- |
| Type-1 | 10.53° | [0 0 0 1] |
| Type-2 | 60° | [1 1 $\bar{\text{2}}$ 0] |
| Type-3 | 60.83° | [$\bar{\text{1.377}}$ $\bar{1}$ 2.377 0.359] |
| Type-4 | 63.26° | [$\bar{\text{10}}$ 5 5 $\bar{\text{3}}$] |
| Type-5 | 90° | [1 $\bar{\text{2.38}}$ 1.38 0] |

**Table S2.** The mechanical properties of the Ti-Cr-Zr-Al-*x*N Ti alloys.

| **Alloys** | **σ_0.2_ (MPa)** | **σ_UTS_ (MPa)** | **ε_u_ (%)** | **ε_f_ (%)** |
| --- | --- | --- | --- | --- |
| LML-AC base alloy-1 | 1106 | 1259 | 4.3 | 12.8 |
| LML-AC base alloy-2 | 1082 | 1216 | 5.1 | 13.2 |
| LML-AC base alloy-3 | 1143 | 1264 | 3.9 | 11.4 |
| LML-AC base alloy-4 | 1065 | 1201 | 4.8 | 13.9 |
| LML-AC base alloy-5 | 1156 | 1275 | 4.5 | 13.1 |
| LML-WQ base alloy-1 | 926 | 1245 | 9.4 | 15.5 |
| LML-WQ base alloy-2 | 885 | 1210 | 10.1 | 16.2 |
| LML-WQ base alloy-3 | 954 | 1275 | 8.9 | 14.3 |
| LML-WQ base alloy-4 | 938 | 1268 | 9.8 | 14.7 |
| LML-WQ base alloy-5 | 873 | 1208 | 10.3 | 15.7 |
| LM-AC 0.4N alloy-1 | 1469 | 1573 | 3.1 | 5.1 |
| LM-AC 0.4N alloy-2 | 1493 | 1615 | 3.7 | 4.2 |
| LM-AC 0.4N alloy-3 | 1481 | 1606 | 4.0 | 5.3 |
| LM-AC 0.4N alloy-4 | 1476 | 1580 | 3.1 | 5.4 |
| LM-AC 0.4N alloy-5 | 1443 | 1566 | 3.5 | 4.4 |
| LM-WQ 0.4N alloy-1 | 1432 | 1658 | 8.6 | 11.4 |
| LM-WQ 0.4N alloy-2 | 1415 | 1661 | 8.3 | 10.8 |
| LM-WQ 0.4N alloy-3 | 1431 | 1634 | 9.8 | 12.4 |
| LM-WQ 0.4N alloy-4 | 1447 | 1640 | 10.1 | 10.7 |
| LM-WQ 0.4N alloy-5 | 1426 | 1644 | 8.4 | 9.8 |
| LML-WQ 0.4N alloy-1 | 1525 | 1869 | 10.2 | 13.1 |
| LML-WQ 0.4N alloy-2 | 1546 | 1872 | 10.4 | 13.0 |
| LML-WQ 0.4N alloy-3 | 1565 | 1896 | 10.8 | 11.2 |
| LML-WQ 0.4N alloy-4 | 1518 | 1862 | 10.5 | 12.1 |
| LML-WQ 0.4N alloy-5 | 1506 | 1845 | 8.7 | 13.2 |

Note: All tests were repeated at least five times to ensure the data reproducibility.

**Table S3.** The mechanical properties of the Ti-Cr-Zr-Al-*x*N Ti alloys.

| **Alloys** | **σ_0.2_ (MPa)** | **σ_UTS_ (MPa)** | **ε_u_ (%)** | **ε_f_ (%)** |
| --- | --- | --- | --- | --- |
| LML-AC base alloy | 1104 ± 16 | 1239 ± 22 | 4.5 ± 0.5 | 12.8 ± 0.9 |
| LML-WQ base alloy | 919 ± 29 | 1241 ± 21 | 9.7 ± 0.6 | 15.2 ± 0.8 |
| LM-AC 0.4N alloy | 1472 ± 18 | 1588 ± 21 | 3.4 ± 0.3 | 4.9 ± 0.6 |
| LM-WQ 0.4N alloy | 1430 ± 12 | 1645 ± 20 | 9.0 ± 0.5 | 11.0 ± 0.9 |
| LML-WQ 0.4N alloy | 1532 ± 23 | 1869 ± 18 | 10.2 ± 0.7 | 12.5 ± 0.8 |

Note: Error bars indicate standard deviations for five tests.

**Table S4.** Phase composition (at %) of the present LML-WQ 0.4N Ti alloys.

| **Phase type** | **Ti** | **Cr** | **Zr** | **Al** | **N** |
| --- | --- | --- | --- | --- | --- |
| α_p_ | 88.6 ± 0.58 | 0.89 ± 0.36 | 1.6 ± 0.28 | 8.5 ± 0.54 | 0.61 ± 0.42 |
| α′ | 85.9 ± 0.86 | 1.9 ± 0.41 | 2.1 ± 0.25 | 8.1 ± 0.67 | 1.6 ± 0.26 |
| β | 83.1 ± 0.95 | 5.1 ± 0.45 | 1.8 ± 0.21 | 7.3 ± 0.35 | 0.72 ± 0.18 |

Note: Phase compositions of LML-WQ 0.4N alloys measured from APT analysis.

**Table S5.** The physical constants used for strength calculation for the LML-WQ 0.4N Ti alloy.

| **Parameter** | **Description** | **α_p_** | **α′** | **β** | **Ref.** |
| --- | --- | --- | --- | --- | --- |
| $K_{L}$ | Hall-Petch constant (MPa·m^-1/2^) | 0.0671 | | | This work |
| *f* | Volume fraction (%) | 27.4 | 49.1 | 23.5 | This work |
| *μ* | Shear modulus (GPa) | 44 | 44 | 39 | ^[13,28]^ |
| *b* | Burgers vector magnitude (Å) | 2.95 | 2.95 | 2.8 | ^[13,28]^ |

**Table. S6.** Measured chemical compositions of the Ti-2.8Cr-4.5Zr-5.2Al-*x*N alloys.

| Alloys | Ti | Cr | | Zr | Al | O | H | C | N |
| --- | --- | --- | --- | --- | --- | --- | --- | --- | --- |
| Base alloy | Bal. | | 2.94 | 4.69 | 5.26 | 0.062 | 0.0041 | 0.012 | 0.005 |
| 0.3N alloy | Bal | | 2.98 | 4.58 | 5.67 | 0.075 | 0.0032 | 0.004 | 0.315 |
| 0.4N alloy | Bal. | | 2.91 | 4.57 | 5.31 | 0.064 | 0.0048 | 0.005 | 0.421 |
| 0.5N alloy | Bal | | 2.86 | 4.82 | 5.46 | 0.082 | 0.0036 | 0.007 | 0.528 |

**Reference:**

[1] T. Zhang, J. Zhu, T. Yang, J. Luan, H. Kong, W. Liu, B. Cao, S. Wu, D. Wang, Y. Wang, C.-T. Liu, A new α + β Ti-alloy with refined microstructures and enhanced mechanical properties in the as-cast state, *Scr. Mater.* **2022**, 207, 114260.

[2] W. Zhu, J. Lei, C. Tan, Q. Sun, W. Chen, L. Xiao, J. Sun, A novel high-strength β-Ti alloy with hierarchical distribution of α-phase: The superior combination of strength and ductility, *Mater. Des.* **2019**, 168, 107640.

[3] G. Lütjering, J. C. Williams, *Titanium*, Springer Science & Business Media, **2007**.

[4] S. Liang, Y. Zhou, L. Yin, Strengthening/Weakening Action of Zr on Stabilizers of Ti Alloys and Its Effect on Phase Transition, *J. Mater. Eng. Perform.* **2021**, 30, 876.

[5] D. Banerjee, J. C. Williams, Perspectives on Titanium Science and Technology, *Acta Mater.* **2013**, 61, 844.

[6] Y. Chong, T. Tsuru, B. Guo, R. Gholizadeh, K. Inoue, N. Tsuji, Ultrahigh yield strength and large uniform elongation achieved in ultrafine-grained titanium containing nitrogen, *Acta Mater.* **2022**, 240, 118356.

[7] H. Conrad, Effect of interstitial solutes on the strength and ductility of titanium, *Prog. Mater. Sci.* **1981**, 26, 123.

[8] Q. Zhao, Q. Sun, S. Xin, Y. Chen, C. Wu, H. Wang, J. Xu, M. Wan, W. Zeng, Y. Zhao, High-strength titanium alloys for aerospace engineering applications: A review on melting-forging process, *Mater. Sci. Eng. A* **2022**, 845, 143260.

[9] Q. Xue, Y. J. Ma, J. F. Lei, R. Yang, C. Wang, Evolution of microstructure and phase composition of Ti-3Al-5Mo-4.5V alloy with varied β phase stability, *J. Mater. Sci. Technol.* **2018**, 34, 2325.

[10] C. de Formanoir, A. Brulard, S. Vivès, G. Martin, F. Prima, S. Michotte, E. Rivière, A. Dolimont, S. Godet, A strategy to improve the work-hardening behavior of Ti–6Al–4V parts produced by additive manufacturing, *Mater. Res. Lett.* **2016**, 5, 201.

[11] X. H. Du, W. P. Li, H. T. Chang, T. Yang, G. S. Duan, B. L. Wu, J. C. Huang, F. R. Chen, C. T. Liu, W. S. Chuang, Y. Lu, M. L. Sui, E. W. Huang, Dual heterogeneous structures lead to ultrahigh strength and uniform ductility in a Co-Cr-Ni medium-entropy alloy, *Nat. Commun.* **2020**, 11, 2390.

[12] T. H. Chou, W. P. Li, H. W. Chang, X. H. Du, W. S. Chuang, T. Yang, Y. T. Zhu, J. C. Huang, Quantitative analysis of hetero-deformation induced strengthening in heterogeneous grain structure, *Int. J. Plast.* **2022**, 159, 103482.

[13] G.-H. Zhao, X. Xu, D. Dye, P. E. J. Rivera-Díaz-del-Castillo, Microstructural evolution and strain-hardening in TWIP Ti alloys, *Acta Mater.* **2020**, 183, 155.

[14] G. H. Zhao, X. Z. Liang, B. Kim, P. E. J. Rivera-Díaz-del-Castillo, Modelling strengthening mechanisms in beta-type Ti alloys, *Mater. Sci. Eng. A* **2019**, 756, 156.

[15] G. S. Dyakonov, S. Mironov, I. P. Semenova, R. Z. Valiev, in *Nanocrystalline Titanium*, (Eds: H. Garbacz, I. P. Semenova, S. Zherebtsov, M. Motyka), Elsevier, **2019**, 123.

[16] S. L. Lu, J. H. Wang, Y. Y. Sun, T. Song, M. Qian, Identification of unusual large zones of Category I triple-alpha-variant clusters in additively manufactured Ti-4Al-2V alloy, *Scr. Mater.* **2022**, 212, 114578.

[17] Y. Chong, T. Bhattacharjee, J. Yi, A. Shibata, N. Tsuji, Mechanical properties of fully martensite microstructure in Ti-6Al-4V alloy transformed from refined beta grains obtained by rapid heat treatment (RHT), *Scr. Mater.* **2017**, 138, 66.

[18] P. Barriobero-Vila, J. M. Vallejos, J. Gussone, J. Haubrich, K. Kelm, A. Stark, N. Schell, G. Requena, Interface-mediated Twinning-induced Plasticity in A Fine Hexagonal Microstructure Generated By Additive Manufacturing, *Adv. Mater.* **2021**, 33, e2105096.

[19] L. Fan, T. Yang, Y. Zhao, J. Luan, G. Zhou, H. Wang, Z. Jiao, C.-T. Liu, Ultrahigh strength and ductility in newly developed materials with coherent nanolamellar architectures, *Nat. Commun.* **2020**, 11, 6240.

[20] D. Caillard, A. Couret, The Hall–Petch law investigated by means of in situ straining experiments in lamellar TiAl and deformed Al, *Microsc. Res. Tech.* **2009**, 72, 261.

[21] X. Wu, Y. Zhu, Heterogeneous materials: a new class of materials with unprecedented mechanical properties, *Mater. Res. Lett.* **2017**, 5, 527.

[22] X. Wu, M. Yang, F. Yuan, G. Wu, Y. Wei, X. Huang, Y. Zhu, Heterogeneous lamella structure unites ultrafine-grain strength with coarse-grain ductility, *Proc. Natl. Acad. Sci. U. S. A.* **2015**, 112, 14501.

[23] W. Li, T.-H. Chou, T. Yang, W.-S. Chuang, J. C. Huang, J. Luan, X. Zhang, X. Huo, H. Kong, Q. He, X. Du, C.-T. Liu, F.-R. Chen, Design of ultrastrong but ductile medium-entropy alloy with controlled precipitations and heterogeneous grain structures, *Applied Materials Today* **2021**, 23, 101037.

[24] S. C. Wang, M. Aindow, M. J. Starink, Effect of self-accommodation on α/α boundary populations in pure titanium, *Acta Mater.* **2003**, 51, 2485.

[25] D. Bhattacharyya, G. B. Viswanathan, R. Denkenberger, D. Furrer, H. L. Fraser, The role of crystallographic and geometrical relationships between α and β phases in an α/β titanium alloy, *Acta Mater.* **2003**, 51, 4679.

[26] E. Farabi, P. D. Hodgson, G. S. Rohrer, H. Beladi, Five-parameter intervariant boundary characterization of martensite in commercially pure titanium, *Acta Mater.* **2018**, 154, 147.

[27] S. L. Lu, C. J. Todaro, Y. Y. Sun, T. Song, M. Brandt, M. Qian, Variant selection in additively manufactured alpha-beta titanium alloys, *J. Mater. Sci. Technol.* **2022**, 113, 14.

[28] C. de Formanoir, G. Martin, F. Prima, S. Y. P. Allain, T. Dessolier, F. Sun, S. Vivès, B. Hary, Y. Bréchet, S. Godet, Micromechanical behavior and thermal stability of a dual-phase α+α’ titanium alloy produced by additive manufacturing, *Acta Mater.* **2019**, 162, 149.
